# Oxygen radical character in group 11 oxygen fluorides

**DOI:** 10.1038/s41467-018-03630-0

**Published:** 2018-03-28

**Authors:** Lin Li, Tony Stüker, Stefanie Kieninger, Dirk Andrae, Tobias Schlöder, Yu Gong, Lester Andrews, Helmut Beckers, Sebastian Riedel

**Affiliations:** 1 Institut für Chemie und Biochemie, Anorganische Chemie, Freie Universität Berlin, Fabeckstrasse 34/36, 14195 Berlin, Germany; 2 Institut für Chemie und Biochemie, Theoretische Chemie, Freie Unversität Berlin, Takustrasse 3, 14195 Berlin, Germany; 3 Physikalisch-Chemisches Institut, Universität Gießen, Heinrich-Buff-Ring 17, 35392 Gießen, Germany; 4 Department of Chemistry, University of Virginia, Charlottesville, VA 22904-431 USA

## Abstract

Transition metal complexes bearing terminal oxido ligands are quite common, yet group 11 terminal oxo complexes remain elusive. Here we show that excited coinage metal atoms M (M = Au, Ag, Cu) react with OF_2_ to form hypofluorites FOMF and group 11 oxygen metal fluorides OMF_2_, OAuF and OAgF. These compounds have been characterized by IR matrix-isolation spectroscopy in conjunction with state-of-the-art quantum-chemical calculations. The oxygen fluorides are formed by photolysis of the initially prepared hypofluorites. The linear molecules OAgF and OAuF have a ^3^*Σ *^−^ ground state with a biradical character. Two unpaired electrons are located mainly at the oxygen ligand in antibonding O−M π* orbitals. For the ^2^*B*_2_ ground state of the OM^III^F_2_ compounds only an O−M single bond arises and a significant spin-density contribution was found at the oxygen atom as well.

## Abstract (web summary)

While transition metal complexes bearing terminal oxido ligands are common, those of group 11 elements have yet to be experimentally observed. Here, Riedel and colleagues synthesise molecular oxygen fluorides of copper, silver and gold, and show that the oxo ligands possess radical character.

## Introduction

Transition metal complexes bearing terminal oxido (O^2−^, oxo) ligands are quite common. Nevertheless, those of the late transition metals are very rare^1–8^, and in particular group 11 terminal oxo complexes (M = Cu, Ag, Au) are to our knowledge still elusive^1,9–12^. On the other side molecular binary group 11 metal oxygen complexes, MO_x_, have extensively been studied in gas phase and matrix-isolation studies^13,14^. Given the well-known activity of silver^15^ and of molecular gold complexes^9,16^ as catalysts in oxygen transfer reactions, there is interest in such molecular group 11 terminal oxo complexes as prospective catalysts in oxidation reactions^17–20^. Copper complexes bearing a terminal oxo ligand were frequently considered as highly reactive intermediates in biological systems to catalyze hydrogen atom transfer and homogeneous oxygenation reactions^11,12^. However, despite experimental and theoretical efforts such proposed group 11 terminal oxo species have never been observed experimentally.

Moreover, computational studies and gas phase experiments revealed an intriguing electronic structure of group 11 metal-oxo complexes^11,17–21^. The diatomic group 11 monoxide cations [MO]^+^ showed a diradical character with two unpaired electrons to reside in antibonding π* orbitals of predominant oxygen 2p character. For [CuO]^+^ this results in a spin density of 1.68 on O and 0.38 on Cu^20,21^. For [AuO]^+^ a higher spin density of 1.92 at oxygen and of only 0.08 at gold indicates a smaller π back-donation from the d orbitals of Au^19^. Triplet ground states have also been predicted quantum mechanically for ligated [LMO]^+^ species in various ligand environments (M = Cu^11,17–19^ and Ag^10^). They are described as M(II) oxido species bearing oxygen centered radicals (O^•−^)^10,17–19^. Consistent with their biradical character these species are found to be highly potent oxidants in conjunction with a very low M-O bond energy. Both, the low M–O bond energy and the high reactivity may have as yet prevented such complexes to be studied in condensed phases.

 Experimental data about molecular oxo complexes of the group 11 metals are extremely rare and simple molecular oxo compounds such as L_n_MO (M = Cu, Ag, Au, L = any ligand except oxygen) are completely unknown.

Here we describe for the first time the synthesis of molecular oxygen fluorides of the coinage metals (L = F). They are characterized by IR matrix-isolation spectroscopy at cryogenic conditions supported by state-of-the-art quantum-chemical calculations. This study gives insight into the nature of the unusual group 11 metal-oxygen bond and proves the radical-like character of the oxo ligands in these complexes. Because of the non-innocent character of the terminal oxo ligand, assigning metal oxidation states in these complexes becomes ambiguous^22–24^.

## Results

### Investigation of hypofluorites

The novel group 11 metal oxygen fluorides have been prepared by laser ablation of the metals in the presence of OF_2_. Laser-ablated metal atoms were allowed to react with either ^16^OF_2_ or ^18^OF_2_ seeded in an excess of argon or neon and the mixture was co-deposited onto a rhodium-plated mirror cooled to 5–15 K (Supplementary Methods). It is expected that the metal atoms insert into the O−F bond of OF_2_ to yield the primary product FMOF, Equation (1).

1 $${\mathrm{M}} ^\ast + {\mathrm{OF}}_2 \to {\mathrm{FMOF}}\,\left( {{\mathrm{M}} = {\mathrm{Cu}},\,{\mathrm{Ag}},\,{\mathrm{Au}}} \right)$$

**Fig. 1:**
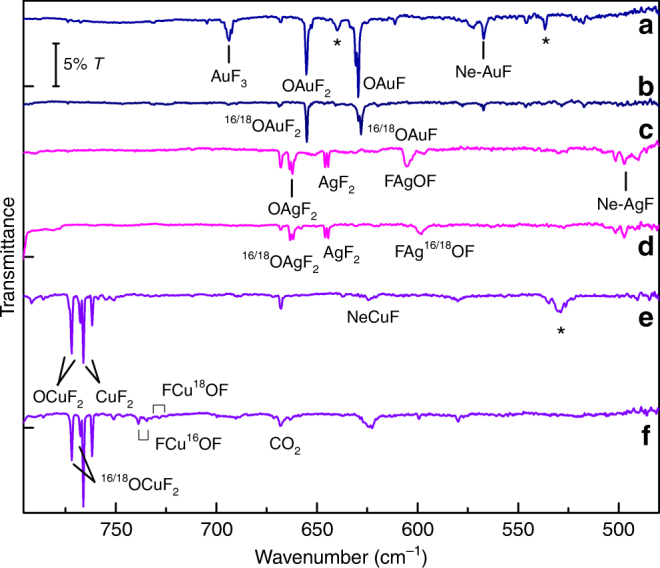
IR Matrix-Isolation Spectra in Neon. IR spectra of the reaction products obtained from laser-ablated Au (**a**, **b**), Ag (**c**, **d**), and Cu (**e**, **f**) with ^16/18^OF_2_ seeded in excess of Ne and co-deposited for 90 min at 5 K. **a** Au+^16^OF_2_ (0.01% in Ne), **b** Au+^18^OF_2_ (0.02% in Ne), **c** Ag+^16^OF_2_ (0.1% in Ne), **d** Ag+^18^OF_2_ (0.1% in Ne), **e** Cu+^16^OF_2_ (0.1% in Ne), **f** Cu+^18^OF_2_ (0.1% in Ne). Assignments are indicated and unassigned features are marked by asterisks. Further spectra are provided in the SI

**Fig. 2:**
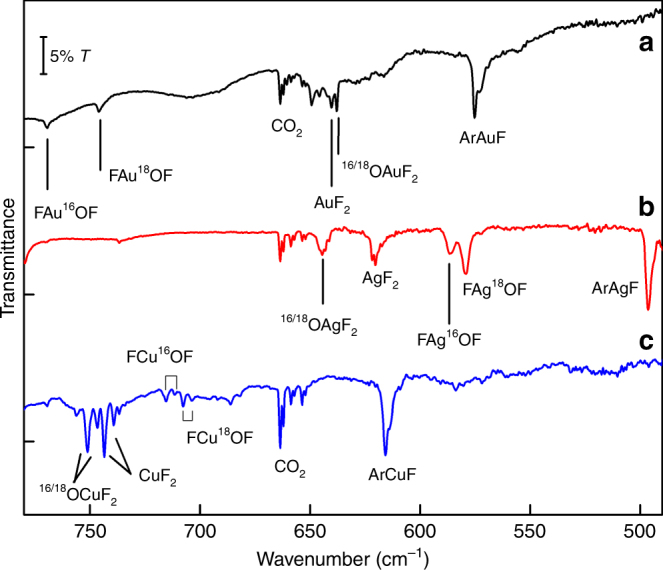
IR Matrix-Isolation Spectra in Argon. IR spectra of the reaction products obtained from laser-ablated Au (**a**), Ag (**b**), and Cu (**c**) with ^16/18^OF_2_ seeded in excess of Ar. **a** Au + ^16/18^OF_2_ (0.5% in Ar, deposited for 110 min at 10 K), **b** Ag + ^16/18^OF_2_ (0.5% in Ar, 60 min at 15K), **c** Cu + ^16/18^OF_2_ (0.5% in Ar, 120 min at 15 K).

The hypofluorites FMOF were indeed detected for all coinage metals, see Figs. 1 and 2 (Supplementary Figures 1–11). Based on their characteristic ^16/18^O isotope shift we unambiguously assigned several stretching bands of these novel species. A compilation of the experimentally observed band positions of oxygen metal fluorides is given in Table 1 together with computed values at the CCSD(T)/aug-cc-pVTZ(-PP) level of theory. For the Cu and Ag species we also observed an additional ^63^Cu/^65^Cu (69:31) and ^107^Ag/^109^Ag (52:48), respectively, isotope splitting according to the natural isotope abundances of these elements. Gold is a neat element and the IR spectra do not show any Au isotope splitting.

**Table 1:** Selected experimental and calculated normal mode frequencies and their ^16/18^O isotope shifts Δν (in cm^–1^) of coinage metal oxo fluoride stretching modes

	Exp. (Ne matrix)			Exp. (Ar matrix)			CCSD(T) Calc. (Int.)^a^	
	ν(^16^O)	ν(^18^O)	Δν	ν(^16^O)	ν(^18^O)	Δν	ν(^16^O)	Δν(^16/18^O)
OAuF	–	–	–	–	–	–	767.9(9)	34.9
	629.4	628.0	1.4	–	–	–	642.6(88)	5.5
OAuF_2_	655.3	655.3	0	634.6	634.6	0	659.2(147)	0
	–	–	–	–	–	–	620.3(6)	0.9
	–	–	–	–	–	–	617.7(0)	32.1
FAuOF	771.4	747.9	23.5	769.5	745.8	23.7	835.4(128)	27.1
	670.4	658.9	11.5	660.6	649.4	11.2	679.7(83)	12.1
	–	–	–	–	–	–	614.1(48)	21.0
O^107^AgF	–	–	–	–	–	–	648.0(13)	9.2
	491.6	472.9	18.7	481.5	463.3	18.2	485.0(155)	18.0
O^109^AgF	–	–	–	–	–	–	646.5(13)	9.1
	491.6	472.9	18.7	481.5	463.3	18.2	485.0(155)	18.0
O^107^AgF_2_	663.6	663.3	0.3	644.6	644.6	0	672.1(152)	0
	–	–	–	–	–	–	576.1(6)	0.1
	–	–	–	–	–	–	497.8(1)	24.9
O^109^AgF_2_	662.3	661.9	0.4	643.0	643.0	0	670.5(152)	0
	–	–	–	–	–	–	576.1(6)	0.1
	–	–	–	–	–	–	497.2(1)	24.8
F^107^AgOF	834.6	811.0	23.6	840.0	815.2	24.8	956.8(230)	28.3
	605.1	598.3	6.8	586.9	578.5	8.4	570.0(56)	1.1
	–	–	–	481.5	463.3	18.2	299.6(114)	14.9
F^109^AgOF	834.6	811.0	23.6	840.0	815.2	24.8	956.8(230)	28.3
	605.1	598.3	6.8	586.9	578.5	8.4	570.0(57)	2.2
	–	–	–	481.5	463.3	18.2	299.5(114)	14.5
O^63^CuF_2_	772.0	772.0	0	751.1	751.1	0	788.8(133)	0
	–	–	–	–	–	–	641.5(4)	0.1
	–	–	–	–	–	–	510.2(2)	23.2
O^65^CuF_2_	767.7	767.7	0	746.5	746.5	0	784.2(132)	0
	–	–	–	–	–	–	641.4(4)	0.1
	–	–	–	–	–	–	509.1(2)	23.6
	–	–	–	–	–		892.2(4)	29.1
F^63^CuOF	736.0	728.5	7.5	715.3	707.7	7.6	761.7(197)	7.0
	–	–	–	–	–	–	602.0(2)	21.1
	–	–	–	–	–	–	891.5(5)	29.1
F^65^CuOF	732.1	724.6	7.5	711.5	704.0	7.5	758.0(195)	6.9
	–	–	–	–	–	–	601.9(2)	21.2

^a^Values calculated at CCSD(T)/aug-cc-pVTZ(-PP) using the CFOUR program. Intensities in [km/mol] are given in parentheses. A more complete set of experimental and calculated frequencies are shown in the Supplementary Tables 1–3

As can be judged from the data in Table 1 the prediction of the band positions of the novel species and even of their ^16/18^O isotope shift is a challenge even for the high CCSD(T) level used therein. In agreement with the CCSD(T) predictions only the Cu−F stretching band of FCuOF has sufficiently high intensity to be observed experimentally. On the contrary, for FMOF, M = Ag and Au, the O−F stretch is predicted to be stronger than the F−M stretches. For both hypofluorites these two stretching bands are clearly assigned. While the O−F stretches show a characteristic Δν(^16/18^O) shift of about 23.5 cm^–1^ (Table 1), the band positions as well as the associated ^16/18^O shift of the F−Ag stretch in FAg^16/18^OF revealed an unusual matrix dependence, as these values were found to be larger in solid Ar than in solid Ne (Table 1). This observation indicates a rather strong interaction of this species with the solid hosts. Furthermore, for the FAg^16/18^OF isotopologues also a strong M−O stretch is computed around 300 cm^–1^ at the CCSD(T) level (Table 1). However, our search for these additional bands in the FIR region failed (Supplementary Figures 6 and 8).

### Investigation of OMF_2_

We noticed a strong dependence of the intensity of the hypofluorite bands on experimental conditions, such as the choice of the matrix gas, its OF_2_ concentration, and the amount of the laser energy used for metal ablation. The FMOF species are found to be highly photo-sensitive. By irradiation of the deposits using visible light of λ > 500 nm they rapidly photo-rearrange to yield the monooxygen fluorides OMF_2_, Equation (2) (Supplementary Figure 2–5, 7, 9, 11).

2 $${\mathrm{FMOF}}\mathop{\longrightarrow}\limits^{{{{h\nu}}}}_{{\lambda > 500\,{\mathrm{nm}}}}{\mathrm{OMF}}_2\quad \left( {{\mathrm{M = Cu,}}\,{\mathrm{Ag,}}\,{\mathrm{Au}}} \right)$$

On the other side, UV or broad-band radiation caused a less selective photo-decomposition of the primarily formed FMOF species to yield in addition to OMF_2_ several secondary products of which the OF radicals, as well as the molecular binary metal fluorides MF_n_ are easily identified. Bands of the OF radical, located at 1031.3/1028.6 cm^–1^ (^16^OF in solid Ne/Ar)^25^ and at 1000.2/997.7 cm^–1^ (^18^OF in Ne/Ar), are always present in the IR spectra of the deposits. Another source of OF radicals is the photo-decomposition of the OF_2_ precursor during the deposition. The laser ablation process not only produces excited metal atoms but also a broad band radiation from the plasma plume. This radiation is probably the reason why the hypofluorites FMOF (M = Cu, Ag, Au) have not been detected in some experiments, especially when using very low OF_2_ concentrations, and why the bands of the OF radical and of the concomitantly formed metal fluorides are always present in the IR spectra. Furthermore, the photo-decomposition of OF radicals^26^ by subsequent near-UV radiation will produce free F and O radicals which exhibit a limited mobility within the solid matrices and initiate secondary reactions with metal species trapped nearby.

### Investigation of binary metal fluorides

Bands associated with the known binary metal fluorides MF_n_ (M = Cu, Ag, Au)^27,28^ are safely assigned in oxygen-free experiments, in which OF_2_ was replaced by gaseous fluorine. In these experiments none of the bands assigned to an oxygen-containing species appeared. Our band positions for the difluorides MF_2_, AuF_3_ and of the rare gas Ng-MF (Ng = Ne, Ar) adducts are listed in Supplementary Tables 1–3. They agree well with previously reported experimental and calculated ones^27,28^. We note that the Ne−CuF complex was previously not detected^28^. Our present results show a weak band at 625.3/623.4 cm^–1^ with a corresponding isotope splitting for a ^63^Cu/^65^Cu species, which we tentatively assign to the elusive Ne−CuF complex (Supplementary Figure 1, 10). The gas-phase value for the ^63^CuF IR band was reported at 614.8 cm^–1^^29^, which is close to a computed value of 613.2 cm^–1^ at the CCSD(T)/aug-cc-pVTZ level^28^.

### Quantum-chemical calculations

**Fig. 3:**
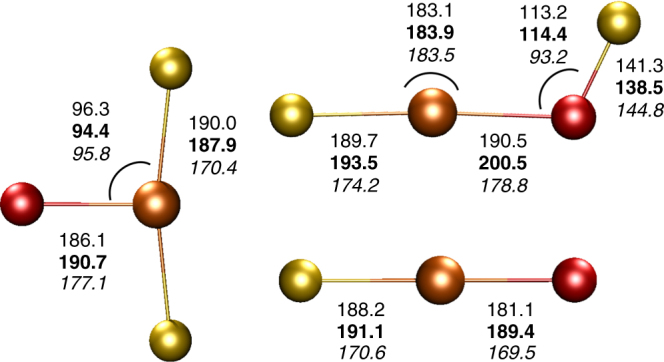
Calculated structures. Structures of OMF_2_, FOMF and OMF (M = Au, Ag and Cu) calculated at the CCSD(T)/aug-cc-pVTZ(-PP) (OMF_2_ and OMF) and at the UB3LYP-D3BJ/def2-TZVPP (FOMF) levels of theory. Bond lengths in pm and angle in degree. Values for M = Au, Ag and Cu are indicated in normal, bold and italic style

Both, the hypofluorites FOMF (*C*_s_ – X ^2^A″) and the oxygen fluorides OMF_2_ (*C*_2v_ – X ^2^B_2_, Fig. 3) have planar structures, and also for the monomolecular FOMF to OMF_2_ rearrangement a planar low-energy transition state has been localized at the UB3LYP-D3BJ/def2-TZVPP level of theory (Supplementary Table 5). The observed fluorine migration from FOMF to OMF_2_ is slightly exothermic for M = Au and Cu by –11.4 and –25.0 kJ mol^−1^, respectively, at the CCSD(T)/augcc-pVTZ level (Supplementary Table 4). For the silver compound FOAgF we computed an endothermic rearrangement to OAgF_2_ by +15.7 kJ mol^−1^, which, however, was photochemically initialized in our experiments.

## Discussion

In accordance with the planar T-shape structures of the OMF_2_ species, featuring an equatorial mono-oxygen ligand and a near linear F−M−F unit (Fig. 3), only the asymmetric M−F stretching frequency was observed for these species (see Figs. 1 and 2 and Table 1). This mode revealed no observable ^16/18^O isotope shift in experiments using mixtures of ^16^OF_2_ and ^18^OF_2_. The close vicinity of these M−F frequencies to the known bands of the corresponding MF_2_ species^27,28^ (Supplementary Tables 1–3) indicates a similar M−F bonding situation. In agreement with the absence of an absorption for the O−M stretches in the recorded spectra a very low intensity is predicted for this mode (Table 1). Both observations indicate an unexpected low polarity of the O−M bond in the OMF_2_ species.

The IR spectra obtained for the gold species in solid Ne (Fig. 1a, b) show another pronounced absorption in the Au−F stretching region at 629.4 cm^–1^ directly after the deposition. This band exhibits a small but significant ^16/18^O shift of 1.4 cm^–1^ in experiments using isotopically enriched ^18^OF_2_ (Table 1), which points to the formation of another so far unknown oxygen containing gold fluoride. Surprisingly, the carrier of this band has not been detected in solid argon, but it is formed in addition to OAuF_2_ in solid neon matrices, for instance by the photo-decomposition of FAuOF using LED light of λ = 375 nm (Supplementary Figure 2c). On the other side, this band was destroyed by visible LED light of λ = 405–455 nm, while simultaneously some OAuF_2_ was formed (Supplementary Figure 1c). These observations suggest that the band at 629.4 cm^–1^ is due to a simple oxygen gold fluoride such as linear OAuF.

Our tentative assignment of the OAuF species is supported by coupled-cluster calculations. We have performed high-level CCSD(T) calculation for the ^3^Σ^–^ ground state of OAuF (*C*_∞v_) using large basis sets (cc-pVQZ, aug-cc-pVQZ)^30,31^. Anharmonicity corrected vibrational frequency calculations via Dunham analysis^32^,  or by the use of a vibrational self-consistent field procedure (VSCF)^33^ (Supplementary Methods). While these calculations predict only one pronounced absorption for ^16^OAuF associated with a strong Au–F stretching mode and a harmonic frequency around 642 cm^–1^, they consistently overestimate its ^16/18^O isotopic shift to about 5 cm^–1^ compared with the experimentally recorded value (1.4 cm^–1^, Table 1). Nevertheless, even this computed ^16/18^O isotopic shift for the Au–F stretching mode of linear OAuF is unusually small, compared to e.g., the linear OAuF unit in FOAuF. The weak coupling between the Au–F and the O–Au stretching vibrations in OAuF indicates an unusual Au–O bond character.

We finally have recorded FIR spectra in the range <500 cm^–1^ using a liquid He cooled bolometer. In the reaction of laser ablated silver atoms with a mixture of ^16^OF_2_ and ^18^OF_2_ (4:6) we observed two additional weak bands at 491.6 and 472.9 cm^–1^ (solid Ne, Supplementary Figure 6; solid Ar: 481.5 and 463.3 cm^–1^, Supplementary Figure 8) showing an unresolved ^107/109^Ag isotope splitting and a significant Δν(^16/18^O) shift of 18.7 (solid Ar: 18.2) cm^–1^. Both, positions and ^16/18^O isotope shift of these bands match very well the CCSD(T) predicted values for the antisymmetric stretching vibrations of the linear triplet ^16/18^OAgF isotopologues (Table 1). Our CCSD(T) calculations also predict a rather strong absorption for the lighter OCuF homolog at 837 cm^–1^ (Δν(^16/18^O) = 22 cm^–1^), which, however, has not been detected in the experimental spectra.

To get more insight into the nature of the M-O bond and the proposed radical-like character of the terminal oxygen ligand in the group 11 complexes we had a closer look on the molecular orbitals and the spin densities for the linear OAuF species (Supplementary Figures 12, 13 and Supplementary Note 1). The O–Au bond length in OAuF of 181.1 pm (Fig. 3), computed at the CCSD(T)/aug-cc-pVTZ(-PP) level of theory, is just in between the reported standard bond length for a single (187 pm)^34^ and double (178 pm)^35^ O–Au bond, while the computed Au–F bond length of 188.2 pm is close to the value expected for an Au–F single bond length of 188 pm^34^. The MO scheme of OAuF bears similarities with that of the valence isoelectronic and well studied [OAuO]^–^ anion^14^. Like the [OAuO]^–^ anion also OAuF shows an open-shell configuration (7σ^2^2π^4^8σ^2^1δ^4^3π^4^9σ^2^4π^2^) with two unpaired electrons in the antibonding 4π* molecular orbitals (MO’s) (Supplementary Table 6 and Figures 12, 13). The key message arising from this scheme is that the oxygen (2p_x_,2p_y_) orbitals, which mainly contribute to the antibonding 4π* MO’s, are higher in energy than the Au 5d orbitals. This is a characteristic feature of a so called inverted ligand field^36–38^, for which the binary group 11 oxides such as the [OAuO]^–^ anion^14^ may serve as an additional example. The computed spin density of 1.34 at oxygen and of 0.53 at gold for triplet OAuF (Supplementary Figure 4) corresponds well to the singly occupied antibonding 4π* MO (Supplementary Figure 13). This bonding pattern is similar to that previously described for the diatomic triplet ^3^[MO]^+^ cations (M = Cu, Au), in which also two biradicaloid π* electrons are present^17–21^. However, while the Au–O bond in the triplet ^3^[AuO]^+^ cation has recently been described by a donor-acceptor model^19^, it is obvious that the Au-O bonding in triplet OAuF is strongly influenced by the bonding properties of the Au 5d orbitals (Supplementary Figures 12, and Supplementary Note 1).

**Fig. 4:**
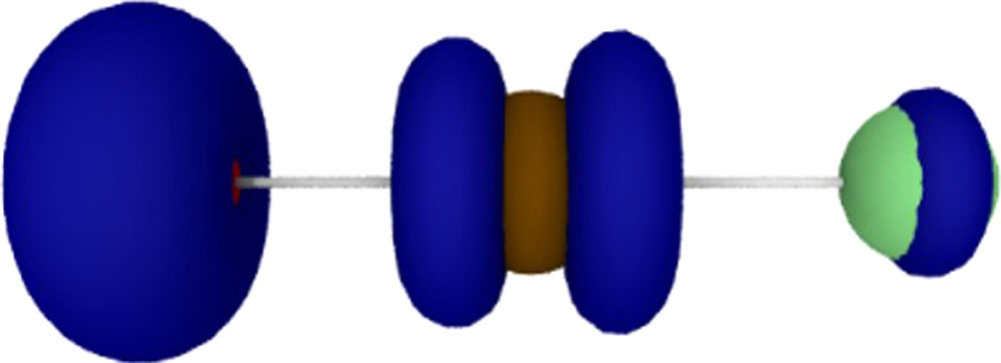
Computed Spin Density. Spin density iso-surface at 0.03 Å^–3^ of OAuF calculated at the DFT B3LYP/aug-cc-pVTZ(-PP) level of theory

Another difference between the Au–O bonds in triplet OAuF and the related ^3^[AuO]^+^ cation is an enhanced spin density at the gold atom (0.53 and 0.08^19^, respectively). There is a significant π back-donation from the Au 5d orbitals in OAuF (Fig. 4) but not for the ^3^[AuO]^+^ cation^19^. This likely can be attributed to the fully occupied F(2p_x_, 2p_y_) orbitals, which in an antibonding fashion mix into the Au(5d_xz_, 5d_yz_) orbitals (Supplementary Figure 13).

In the above described experiments the elusive OMF complexes (M = Au, Ag) are likely formed from free O atoms and MF molecules during deposition or within the solid matrices. These species were always present in the deposits. As it was shown by us, AuF forms considerable strong Ar complexes (Δ*H* = −47.3 kJ mol^−1^), but only weak complexes with neon (Δ*H* = −8.8 kJ mol^−1^)^27,28^. It is therefore reasonable that this reaction was observed using neon matrices by substitution of a weakly bound Ne atom in a NeAuF complex, but not in solid argon where the ArAuF complex was found to be one of the main reaction products (Fig. 2). The elusive OMF (M = Cu, Ag, Au) compounds bear also some similarities to the weakly-bound NeMF adducts, also recently studied by us^28^. While the NeCuF adduct (Δ*H* = −11.2 kJ mol^−1^)^28^ is predicted by CCSD(T) calculations to be even more stable than NeAuF, attempts to observe this elusive species failed until it could now tentatively be assigned in this work. On the other side, the NgAgF (Ng = Ne, Ar) show significantly smaller bonding energies ((Δ*H* = −3.7 (Ne) and −21.1 (Ar) kJ mol^−1^)^28^, but both, the neon and the oxygen complexes of AgF are detected experimentally. Like FOAgF, but unlike OAuF, the elusive OAgF complex is highly photosensitive and easily destroyed by irradiation of visible light (Supplementary Figures 6, 8).

Also in the ^*2*^*B*_2_ ground state of the OMF_2_ species the unpaired electron occupies an oxygen 2p orbital, and the computed spin-density is solely located at the oxygen atom (Supplementary Figure 14). There is no O−M π bond, which is also evident from the computed CCSD(T) O−M bond length indicated in Fig. 3. As pointed out by a referee we noticed that such a breakdown of the conventional Lewis electron–pair model is not a unique phenomenon of group 11 oxygen compounds. One closely related example is the recently investigated oxygen-centered OHgF radical^39^, but also other transition metal terminal oxo complexes bearing terminal oxygen-centered radicals have been predicted^22–24,40^. Terminal oxygen radicals have also been found to occur in metal oxide clusters of early transition metals, when these metals are in their highest oxidation state. Such oxide clusters were found to be crucial intermediates in catalytic oxidation reactions^41,42^.

We have shown that excited coinage metal atoms M (M = Au, Ag, Cu) react with OF_2_ to yield the hypofluorites FOMF, as well as the elusive group 11 oxygen fluorides OAgF, OAuF, and OMF_2_. The isolation of these novel species in rare-gas matrices allowed a direct spectroscopic investigation of group 11 compounds bearing a terminal oxygen ligand in a condensed phase. The new oxygen fluorides are likely formed by photolysis of the initially prepared hypofluorites. The linear molecules OAgF and OAuF have a ^3^*Σ*^−^ ground state with a biradical character. Two unpaired electrons are located mainly at the oxygen ligand in antibonding O–M π* orbitals. For the ^2^*B*_2_ ground state of the OMF_2_ compounds only an O–M single bond arises and a significant spin-density contribution was found at the oxygen atom as well. The radical character of the terminal oxygen ligand in these group 11 fluorides can be explained with an inverted ligand field, which previously has also been suggested for the related binary group 11 oxides^14,19,21^. It turns out that the investigation of these novel oxygen fluorides is a challenge for prospective experimental and high-level quantum chemical studies.

## Methods

### Experimental details

The experimental set-up used a closed-cycle helium cryostat (Sumitomo Heavy Industries, RDK-205D) inside a vacuum chamber (Supplementary Methods). The Nd:YAG laser fundamental with a pulse energy of up to 50 mJ/cm² was focused onto the metal targets. The OF_2_ and ablated metal atoms and their reaction products have been trapped in rare-gas matrices on a cold rhodium-plated mirror. ^16/18^OF_2_ was synthesized by a known procedure using elemental fluorine and ^16/18^OH_2_ dispersed in solid KF. FTIR spectra were recorded on a Bruker Vertex 80 v spectrometer at a 0.5 cm^−1^ resolution and with a 0.5 cm^−1^ accuracy using an MCTB detector (Supplementary Methods). For quantum-chemical details see also Supplementary Methods, Supplementary Table 7 and Supplementary Data 1.

### Data availability

Data will be available on request from the authors.

## Electronic supplementary material

Supplementary Information (PDF 1435 kb)

Description of Additional Supplementary Files(PDF 167 kb)

Supplementary Data 1 (PDF 336 kb)
