# Supplementary material for: Oxygen radical character in group 11 oxygen fluorides
Source: Nat Commun. 2018 Mar 28;9:1267. doi: 10.1038/s41467-018-03630-0 (PMC5871800; doi:10.1038/s41467-018-03630-0)
Supplement: Supplementary file 1 — Supplementary Information (PDF 1435 kb) [file 41467_2018_3630_MOESM1_ESM.pdf]

Oxygen Radical Character in Group 11 Oxygen Fluorides

Lin Li et al.

Supplementary Figure

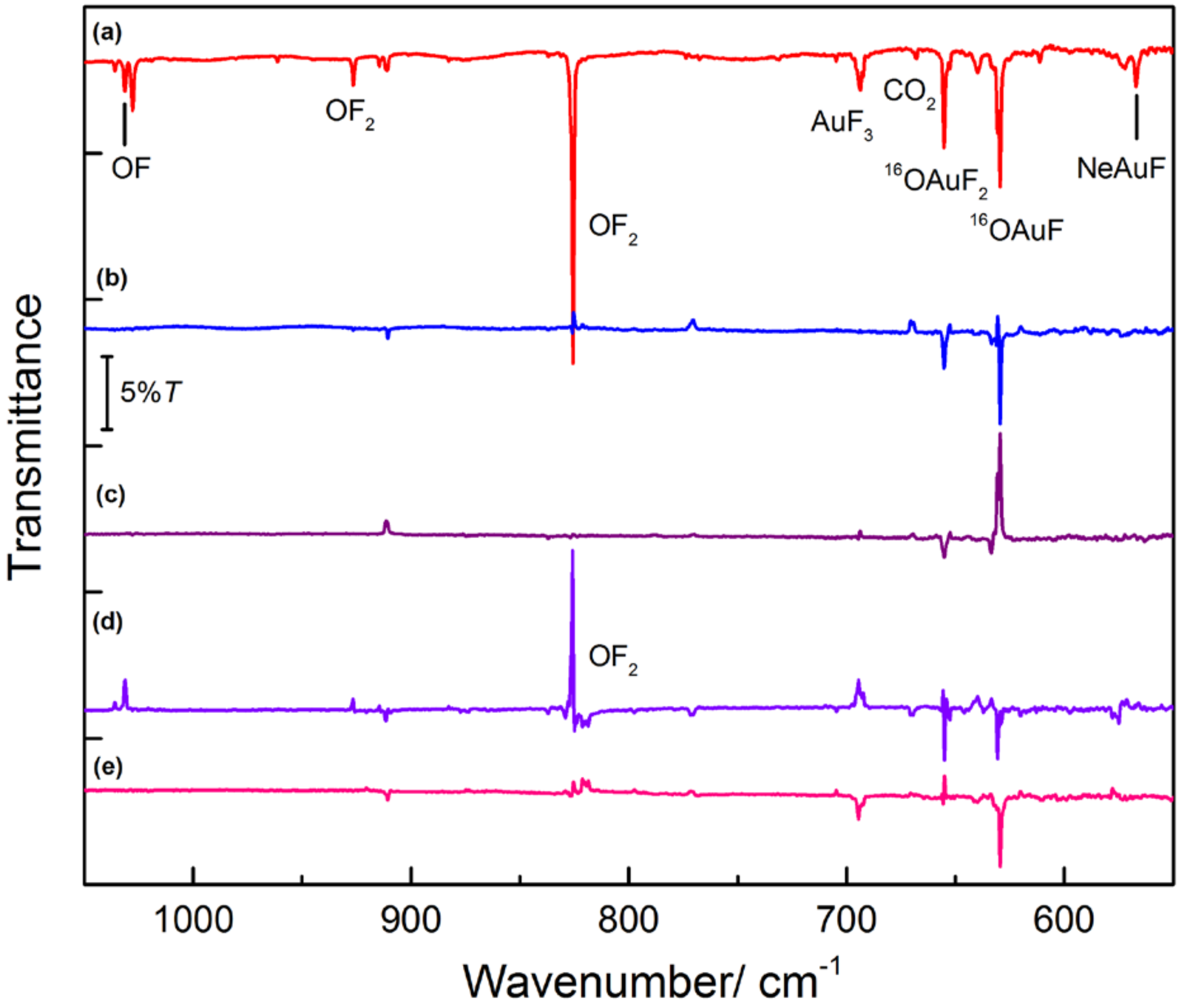

Supplementary Figure 1 Reaction of laser-ablated Au atoms with OF_2 in neon
IR spectra of products obtained from the reaction of laser-ablated Au atoms with $^{16}OF_2$ in excess of neon and co-deposited for 90 min at 5 K: a) Au + $^{16}OF_2$ (0.01% in Ne); b-e) difference spectra recorded after UV 375 nm irradiation for 10 min (b), after UV 405-455 nm (LED) irradiation for 10 min (c), after annealing to 10 K (d), and after subsequent λ=266 nm laser irradiation for 10 min (e). Assigned bands are indicated.

Observations after selective irradiations and annealing of the matrix:

Species	UV 375 nm	Laser 266 nm	UV 405-455 nm	Annealing 10 K
OAuF	Increase	-	Decrease	Increase
$OAuF_2$	Increase	Decrease	-	-
AuF_3	-	Increase	-	Decrease

The band due to $OAuF_2$ at 655.3 cm^{-1} in solid Ne increases after irradiation at λ = 375 nm together with the band recorded at 629.5 cm^{-1} associated with OAuF. Photolysis using laser light at λ = 266 nm only increases the band of AuF_3 (693.8 cm^{-1}) at the expense of $OAuF_2$ at 655.3 cm^{-1} (Supplementary Figure 1). Additional irradiation using LED light of λ = 405-455 nm drastically decreases the band at 629.5 cm^{-1}.

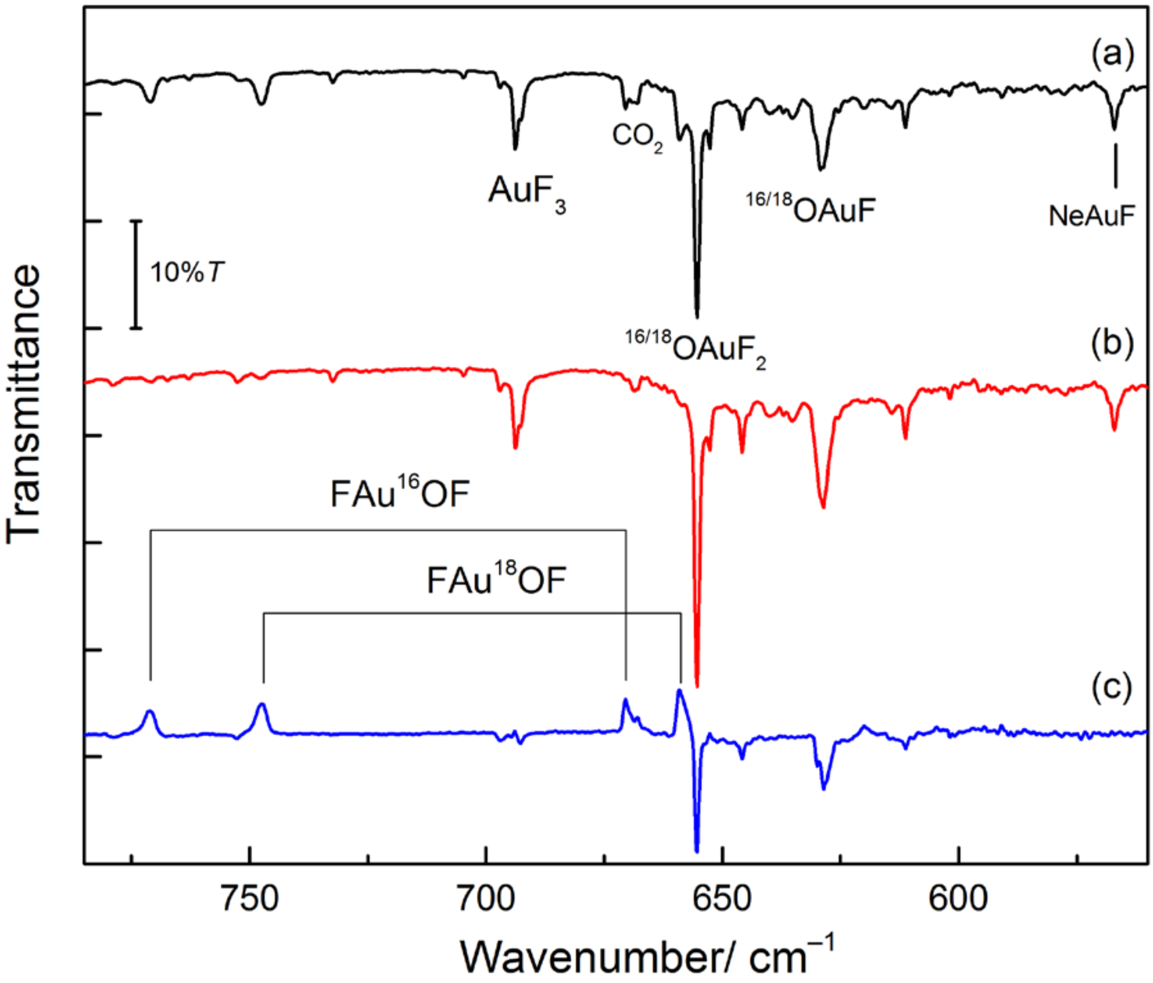

Supplementary Figure 2 Reaction of laser-ablated Au atoms with $^{16/18}OF_2$ in neon
IR spectra of products obtained from the reaction of laser-ablated Au atoms with a mixture $^{16}OF_2$: $^{18}OF_2$ (around 1:1) in excess of neon and co-deposited for 90 min at 7 K: a) Au + $^{16/18}OF_2$ (0.05% in Ne), b) after UV-375 nm irradiation for 15 min, and c) difference spectrum recorded after UV 375 nm irradiation for 25 min.

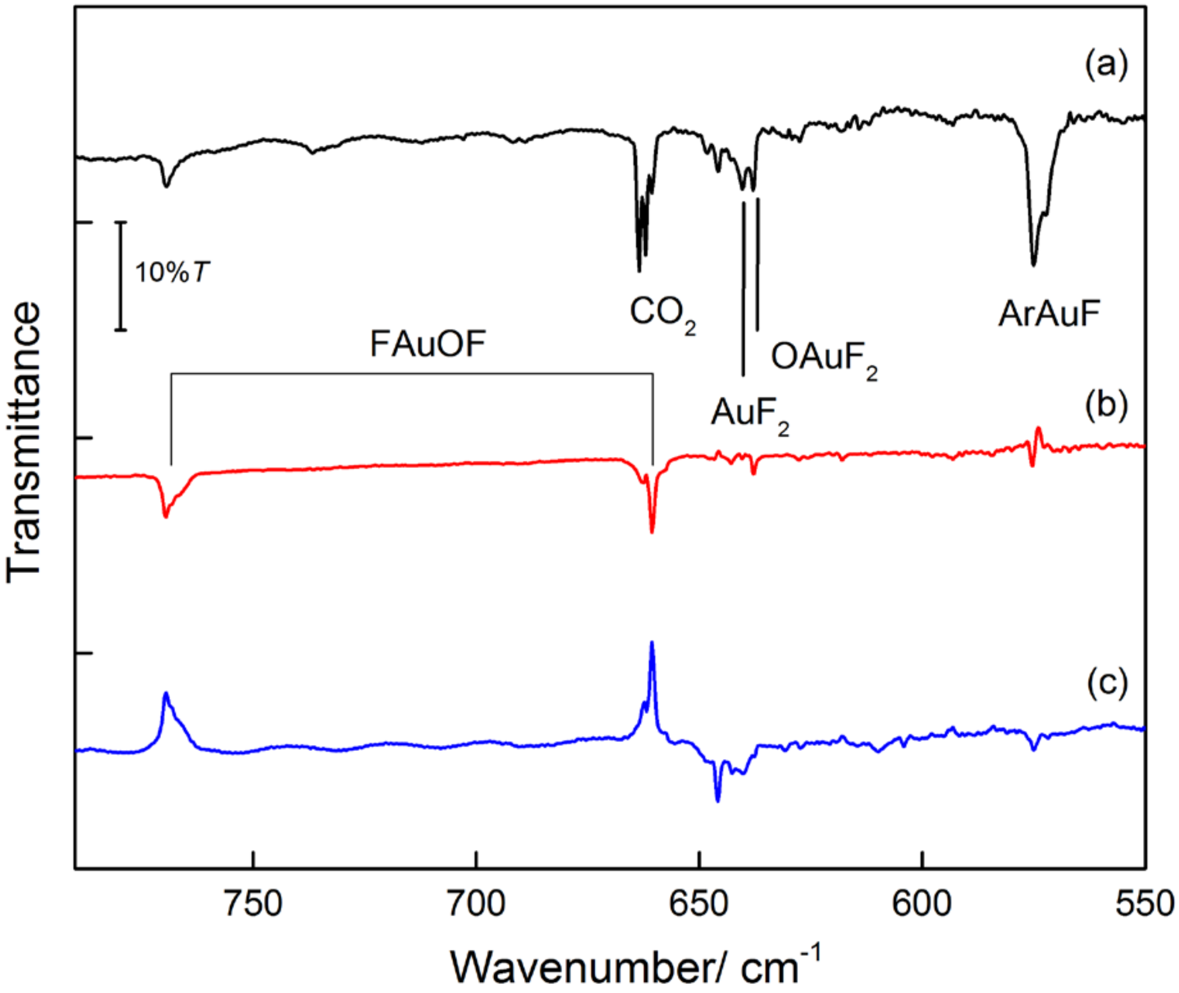

Supplementary Figure 3 Reaction of laser-ablated Au atoms with OF_2 in argon
IR spectra of products obtained from the reaction of laser-ablated Au atoms with $^{16}OF_2$ in excess of argon and co-deposited for 110 min at 10 K: a) Au + $^{16}OF_2$ (0.5% in Ar); b-c) difference spectra measured after annealing at 20 K (b), and after λ = 455 nm (LED) irradiation for 20 min (c).

Observations after selective irradiations and annealing of the matrix:

Species	UV 405-455 nm	UV 375 nm	Laser 266 nm
OAuF	Decrease	Increase	Increase
$OAuF_2$	Increase	Increase	Decrease
FAuOF	Decrease	Decrease	-

Decrease of bands due to FAuOF was observed by irradiation of the deposit using light of λ = 405-455 nm and λ = 375 nm (Supplementary Figures 2 and 3). The band due to $OAuF_2$ at 655.3 cm^{-1} in solid Ne (Supplementary Figure 1) increases after irradiation at λ = 375 nm together with the band recorded at 629.4 cm^{-1} associated with OAuF.

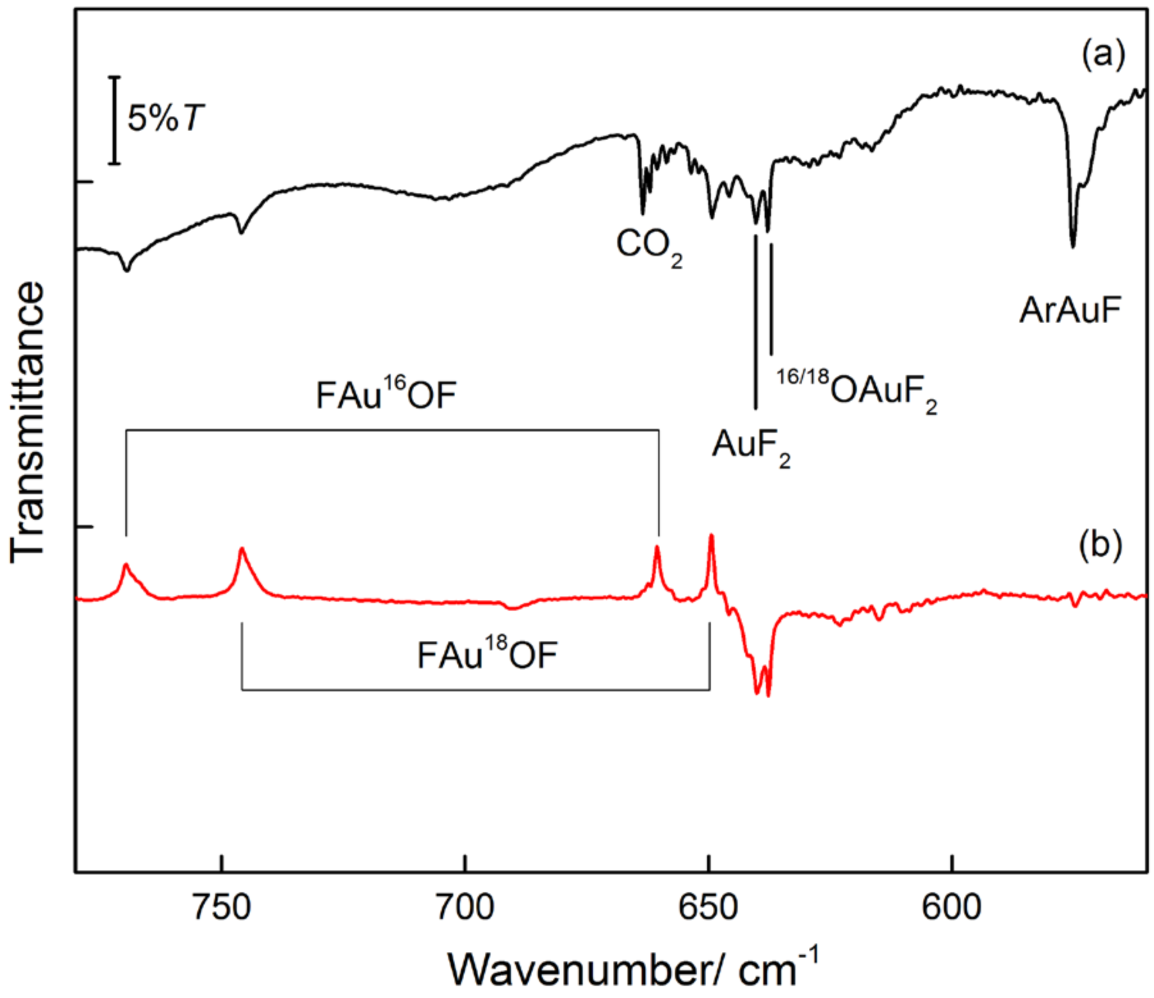

Supplementary Figure 4 Reaction of laser-ablated Au atoms with $^{16/18}OF_2$ in argon

IR spectra of products obtained from the reaction of laser-ablated Au atoms with a mixture $^{16}OF_2$: $^{18}OF_2$ (about 4:6) in excess of argon and co-deposited for 60 min at 15 K: a) Au + $^{16/18}OF_2$ (0.5% in Ar), and b) difference spectrum observed after λ > 280 nm (edge filter) irradiation for 20 min.

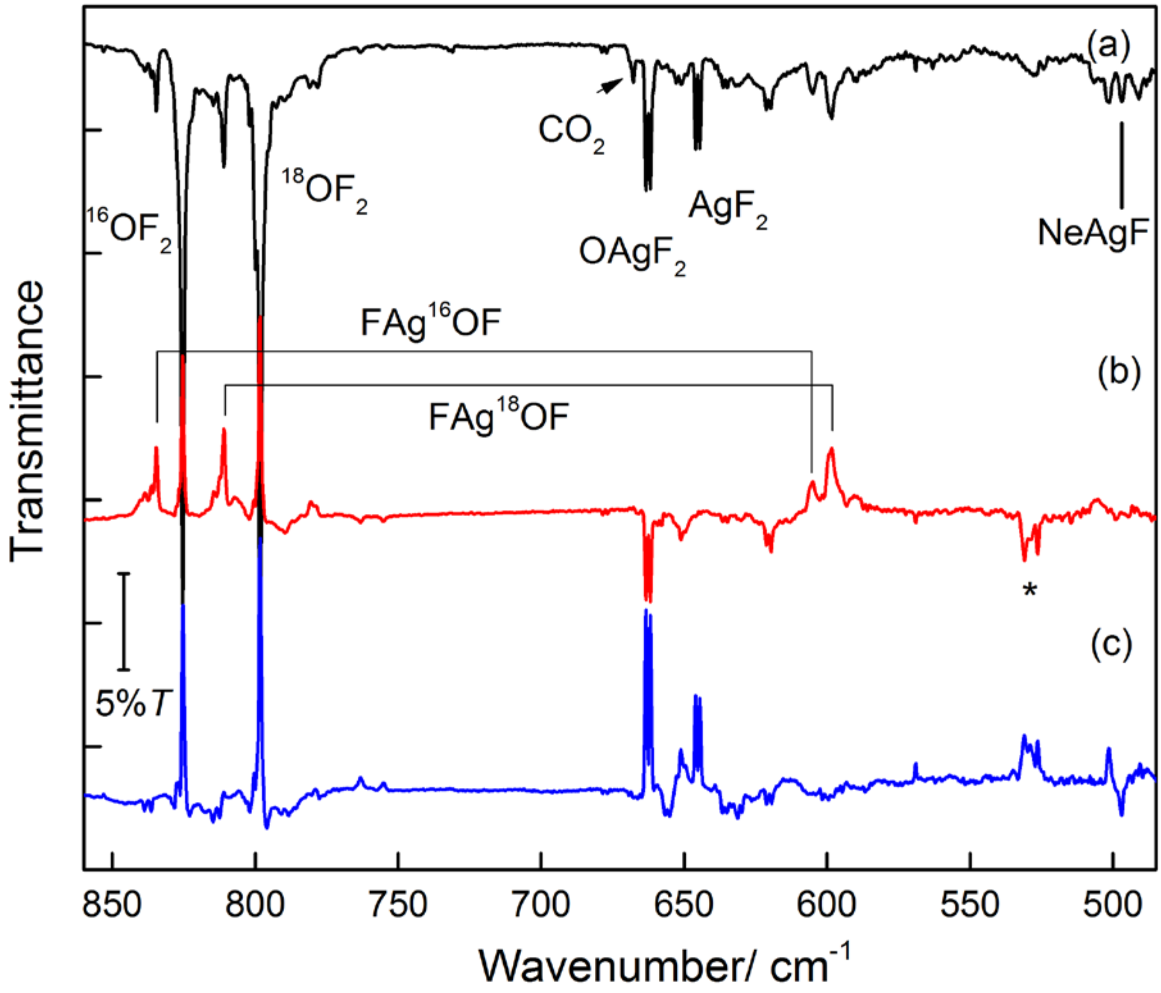

Supplementary Figure 5 Reaction of laser-ablated Ag atoms with OF_2 in neon

IR spectra of products obtained from the reaction of laser-ablated Ag atoms with a mixture $^{16}OF_2$: $^{18}OF_2$ (around 4:6) in excess of neon and co-deposited for 120 min at 7 K: a) Ag + $^{16/18}OF_2$ (0.05% in Ne); b-c) difference spectra observed after λ = 528 nm (LED) irradiation for 15 min (b); and after λ = 455 nm (LED) irradiation for 40 min. Assigned bands are indicated and unassigned bands are marked by an asterisk.

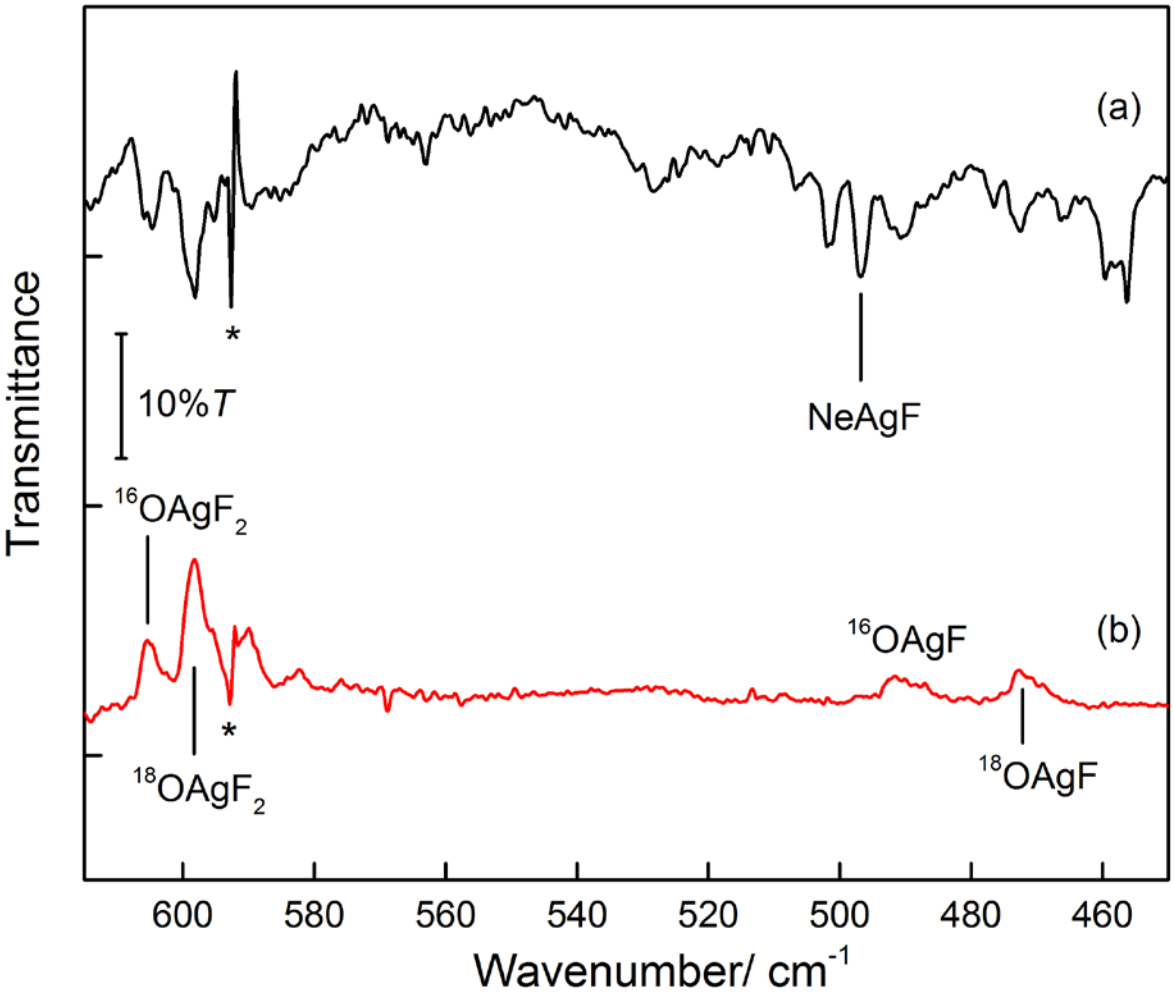

Supplementary Figure 6 Reaction of laser-ablated Ag atoms with $^{16/18}OF_2$ in neon

FIR spectra of products obtained from the reaction of laser-ablated Ag atoms with a mixture $^{16}OF_2$: $^{18}OF_2$ (around 4:6) in excess of neon and co-deposited for 210 min at 7 K: a) Ag + $^{16/18}OF_2$ (0.1% in Ne); b) difference spectra observed after λ = 590 nm (LED) irradiation for 20 min. Asterisks indicate noise of the bolometer.

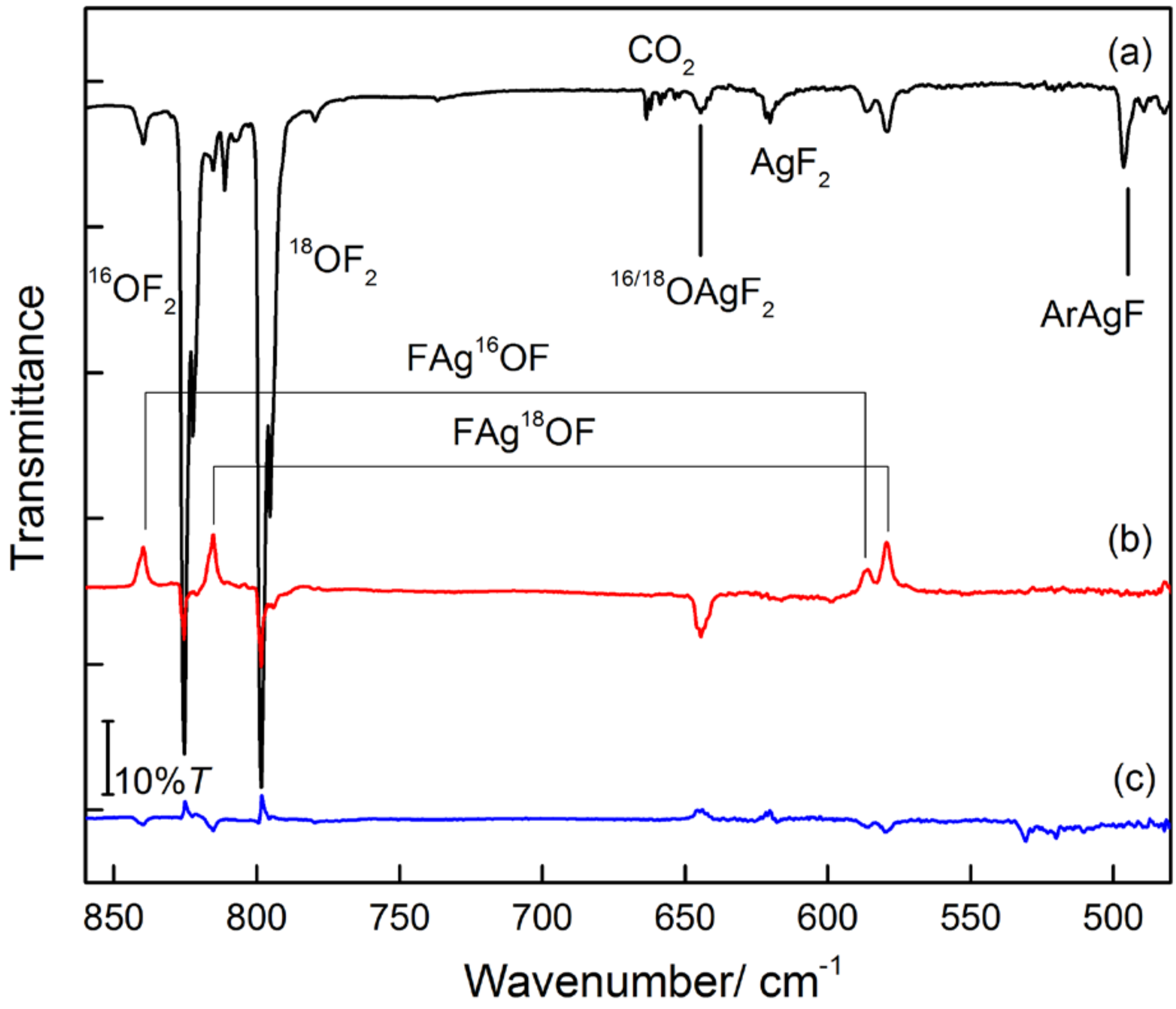

Supplementary Figure 7 Reaction of laser-ablated Ag atoms with $^{16/18}OF_2$ in argon
IR spectra of products obtained from the reaction of laser-ablated Ag atoms with a mixture $^{16}OF_2$: $^{18}OF_2$ (around 4:6) in excess of argon and co-deposited for 60 min at 15 K: a) Ag + $^{16/18}OF_2$ (0.5% in Ar); b-c) difference spectra recorded after λ = 590 nm (LED) irradiation for 15 min (b), and after UV 375 nm irradiation for 25 min (c).

Observations after selective irradiations of the matrix:

Species	590 nm	528 nm	UV 375 nm
OAgF	destroyed	-	-
$OAgF_2$	Increase	Increase	Decrease
AgF_2	-	-	Decrease
FAgOF	destroyed	destroyed	Increase

Irradiation of the deposit using LED light of λ = 590 nm and 528 nm lead to a decomposition of FAgOF while $OAgF_2$ forms, see Supplementary Figure 7. Using UV light of λ = 375 nm some $OAgF_2$ and AgF_2 decomposed while FAgOF was formed simultaneously (Supplementary Figure 7).

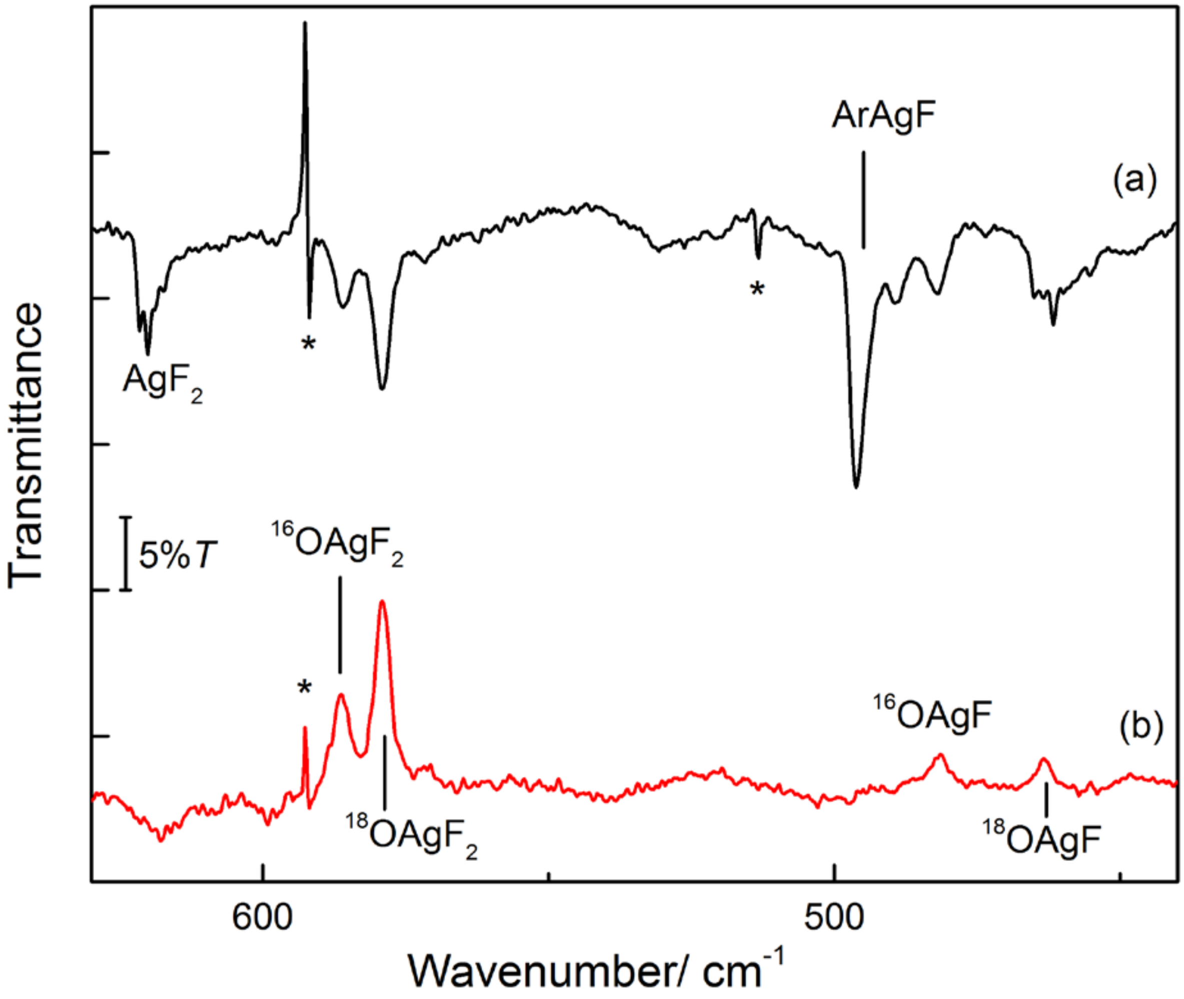

Supplementary Figure 8 Reaction of laser-ablated Ag atoms with $^{16/18}OF_2$ in argon

FIR spectra of products obtained from the reaction of laser-ablated Ag atoms with a mixture $^{16}OF_2$: $^{18}OF_2$ (around 4:6) in excess of argon and co-deposited for 120 min at 15 K: a) Ag + $^{16/18}OF_2$ (0.5% in Ar), and b) difference spectrum observed after λ = 590 nm (LED) irradiation for 15 min. Asterisks indicate noise of the bolometer.

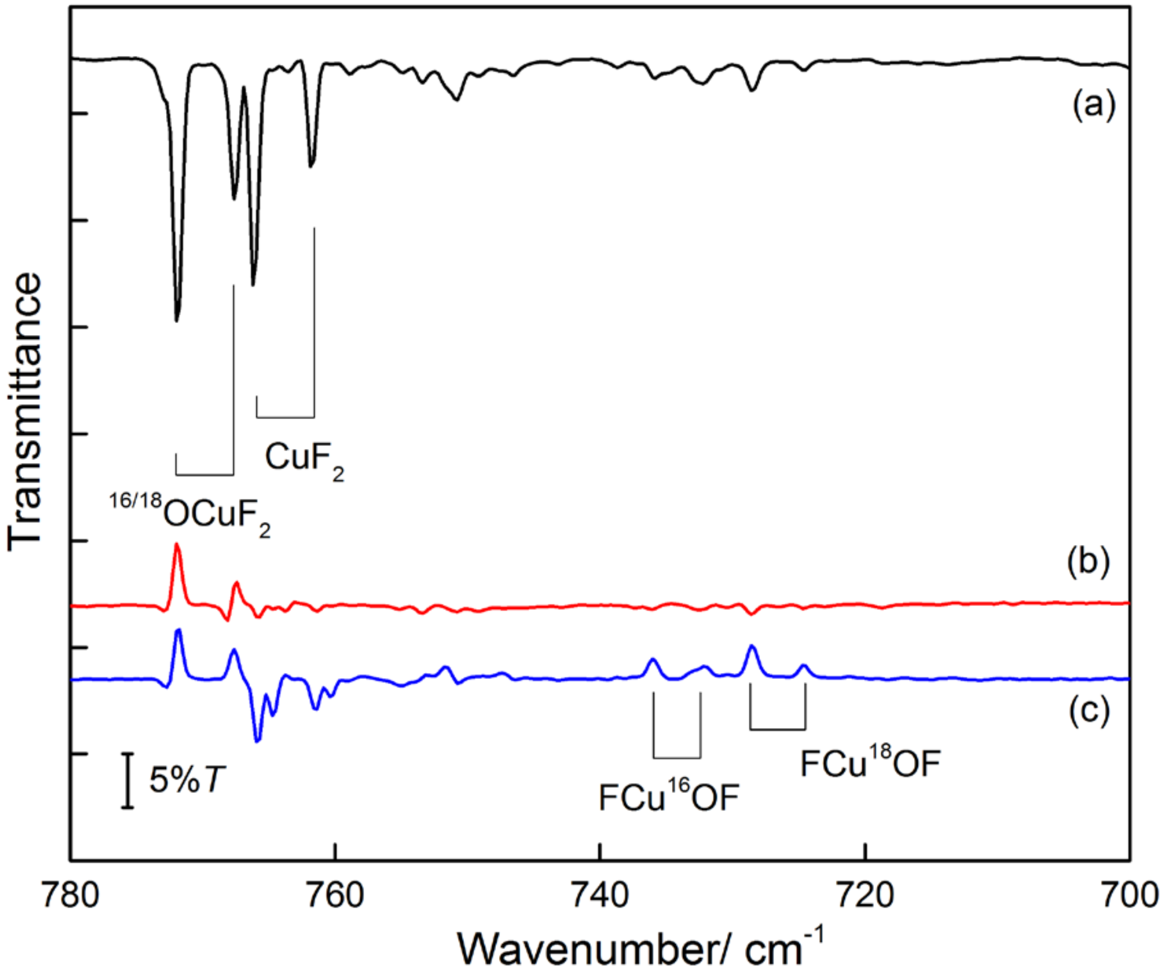

Supplementary Figure 9 Reaction of laser-ablated Cu atoms with $^{16/18}OF_2$ in neon
IR spectra of products obtained from the reaction of laser-ablated Cu atoms with a mixture $^{16}OF_2$: $^{18}OF_2$ (around 4:6) in excess of neon and co-deposited for 120 min at 7 K: a) Cu + $^{16/18}OF_2$ (0.05% in Ne); b-c) difference spectra recorded after λ = 590 nm (LED) irradiation for 10 min (b), and after λ = 528 nm (LED) irradiation for 15 min (c).

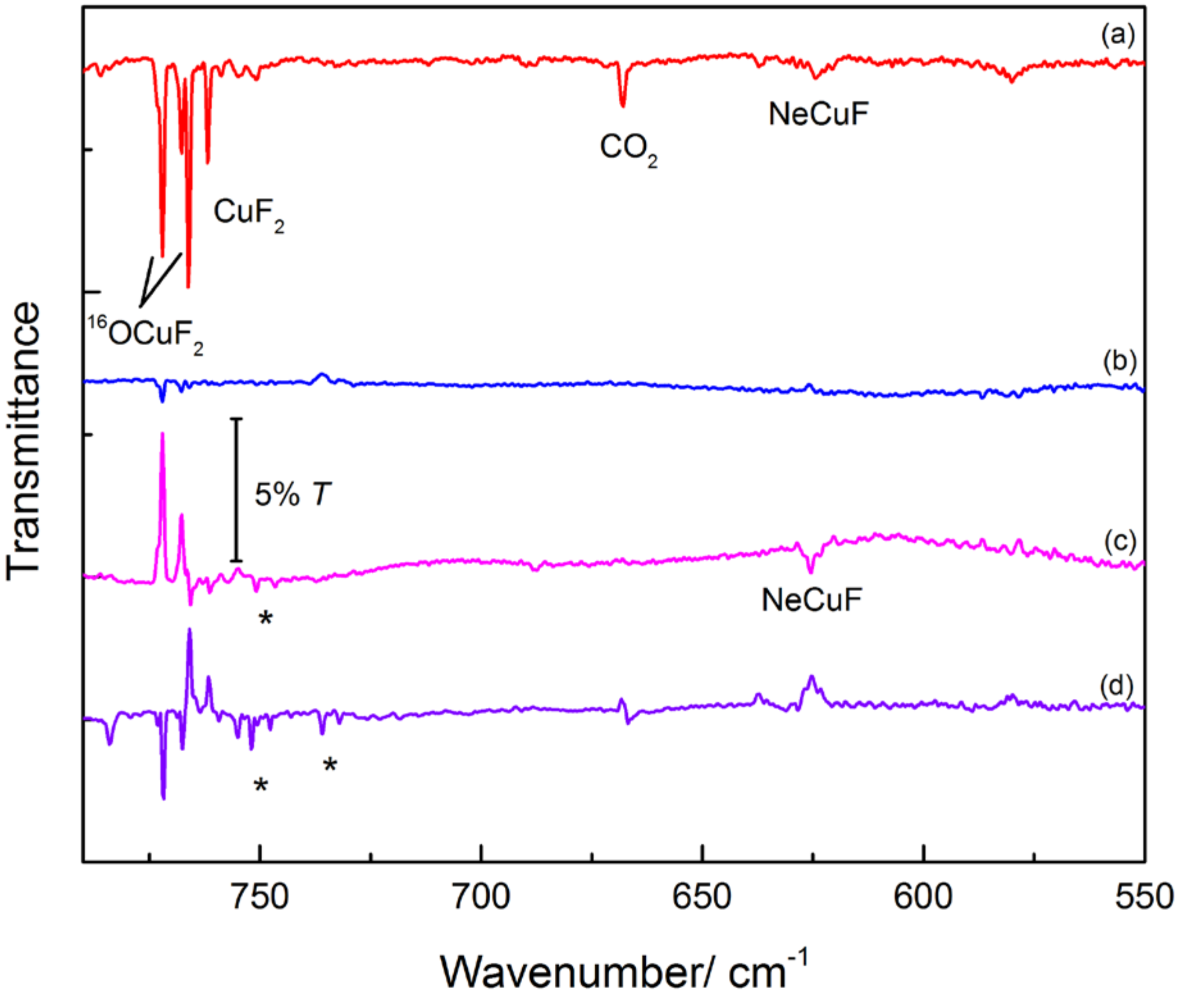

Supplementary Figure 10 Reaction of laser-ablated Cu atoms with OF_2 in neon

IR spectra of products obtained from the reaction of laser-ablated Cu atoms with $^{16}OF_2$ in excess of neon and co-deposited for 90 min at 5 K: a) Cu + $^{16}OF_2$ (0.1% in Ne); b-d) difference spectra observed after UV 375 nm irradiation for 10 min (b), after λ = 266 nm laser irradiation for 10 min (c), and after subsequent annealing to 8 K (d). Assigned bands are indicated and unassigned bands are marked by an asterisk.

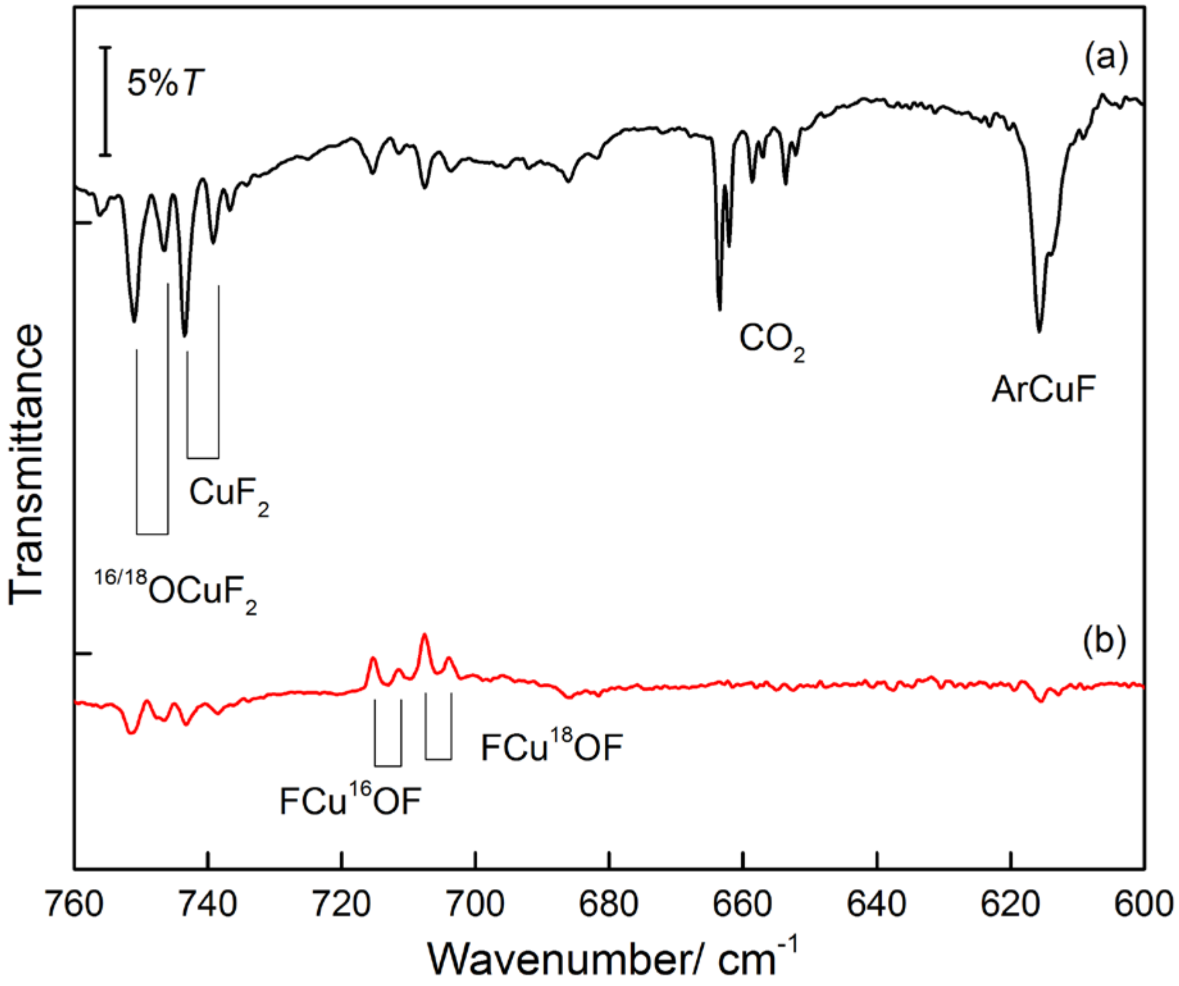

Supplementary Figure 11 Reaction of laser-ablated Cu atoms with $^{16/18}OF_2$ in argon
IR spectra of products obtained from the reaction of laser-ablated Cu atoms with a mixture $^{16}OF_2$: $^{18}OF_2$ (around 4:6) in excess of argon and co-deposited for 120 min at 15 K: a) Cu + $^{16/18}OF_2$ (0.5% in Ar), and b) difference spectrum after λ > 420 nm (edge filter) irradiation for 15 min.

Observations after selective irradiations and annealing of the matrix:

Species	528 nm	Laser 266 nm	420 nm
$OCuF_2$	Decrease	Decrease	Increase
FCuOF	Decrease	Increase	Decrease
CuF_2	Increase	Increase	Increase

Using LED light of λ = 528 nm leads to a decomposition of $OCuF_2$ and FCuOF while CuF_2 increases. The bands due to $OCuF_2$ and CuF_2 increase during photolysis at λ = 375 nm.

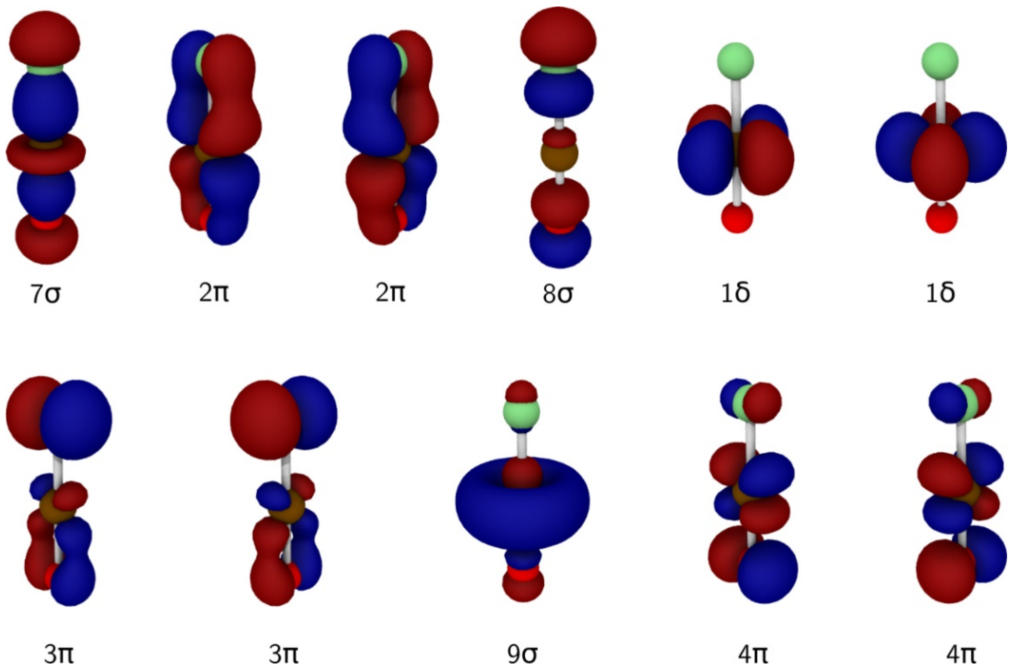

Supplementary Figure 12 Molecular orbitals of OAuF computed at the B3LYP/aug-cc-pVTZ level.

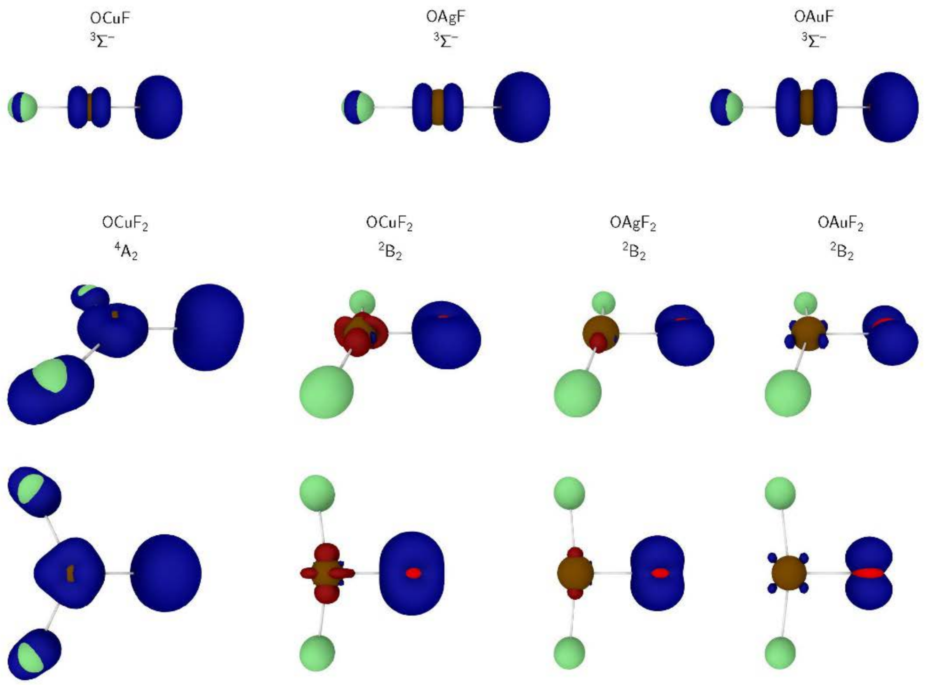

Supplementary Figure 13 Computed spin-density iso-surfaces of OMF and OMF_2 (M = Cu, Ag, Au) at 0.03 Å^{-3}, calculated at the B3LYP/aug-cc-pVTZ level of theory.

Supplementary Tables

Supplementary Table 1 Experimental and calculated frequencies of gold fluorides and oxygen fluorides[a]

	Exp. (this work)		Exp. Ref.[b]		Calc.				
	(Ne)	(Ar)	(Ne)	(Ar)	B3LYP[d]	Int.	CCSD(T)[e]	Int.	CCSD(T)[f]
Ne-AuF	567.1	-	567.2	-	541[c]	47	558.5[b]	-	-
Ar-AuF	-	575.2	-	575.1	559[c]	54	580.7[b]	-	-
^{16}OAuF	629.4	-	-	-	628.5	82	642.4	88	639.2
	-	-	-	-	757.7	3	767.9	9	771.4
^{18}OAuF	628.0	-	-	-	623.6	77	637.1	80	633.8
	-	-	-	-	722.9	7	733.0	17	736.4
AuF_2	-	640.1	664.8	640.1	667[c]	91	647[c]	-	-
	-	-	-	-	593[c]	0	616[c]	-	-
$^{16}OAuF_2$	655.3	634.6	-	-	637.5	141	659.2	147	659.2
	-	-	-	-	632.5	1	620.3	6	621.4
	-	-	-	-	579.8	5	617.7	0	616.0
$^{18}OAuF_2$	655.1	634.6	-	-	637.4	147	659.2	147	659.2
	-	-	-	-	599.9	1	619.4	6	619.7
	-	-	-	-	596.7	5	585.6	0	584.7
FAu^{16}OF	771.4	769.5	-	-	894.2	173	835.4	128	845.0
	670.4	660.6	-	-	654.2	58	679.7	83	688.5
	-	-	-	-	586.6	34	614.1	48	628.9
FAu^{18}OF	747.9	745.8	-	-	866.4	161	808.3	118	816.1
	658.9	649.4	-	-	650.0	74	667.6	107	671.6
	-	-	-	-	563.7	19	593.1	24	612.9
AuF_3	692.5	690.4	692.4	690.1	643[c]	151	669.5[c]	-	-
	-	-	-	-	614[c]	4	634.6[c]	-	-
	-	-	-	-	601[c]	14	612.2[c]	-	-

[a] Frequencies in [cm^{-1}] and intensities in [km/mol]. **[b]** X. Wang, L. Andrews, K. Willmann, F. Brosi, S. Riedel, *Angew. Chem. Int. Ed.* **2012**, *51*, 10628. Values are computed at CCSD(T)/aug-cc-pVTZ(-PP) level. **[c]** X. Wang, L. Andrews, F. Brosi, S. Riedel, *Chem. Eur. J.* **2013**, *19*, 1397. Values calculated at B3LYP/aug-cc-pVTZ(-PP) and CCSD(T)/aug-cc-pVTZ(-PP) level. **[d]** This work, calculated at B3LYP/aug-cc-pVTZ(-PP) level. **[e]** This work, values calculated at CCSD(T)/aug-cc-pVTZ(-PP) using the CFOUR program. **[f]** This work, values computed at CCSD(T)/aug-cc-pVTZ(-PP) level using the MOLPRO program package.

Supplementary Table 2 Experimental and calculated frequencies of silver fluorides and oxygen fluorides[a]

	Exp. (this work)		Exp Ref.[b]		Calc.			
	(Ne)	(Ar)	(Ne)	(Ar)	B3LYP[c]	Int.	CCSD(T)[c]	Int.
Ne-^{107}AgF	497.0	-	497.3	-	496[b]	53	507.6[b]	-
Ne-^{109}AgF	-	-	-	-	-	-	506.9[b]	-
Ar-^{107}AgF	-	497.6	-	497.2	505[b]	61	518.5[b]	-
Ar-^{109}AgF	-	-	-	-	-	-	517.7[b]	-
$^{107}AgF_2$	646.1	621.7	646.1	621.7	620.9[b]	38	653.1[b]	-
$^{109}AgF_2$	644.5	620.2	644.5	620.0	619.4[b]	38	651.5[b]	-
$^{107}AgF_3$	-	-	679.0	661.9	667.0[b]	133	689.5[b]	-
$^{109}AgF_3$	-	-	677.5	660.4	665.2[b]	133	687.8[b]	-
$^{16}O^{107}AgF$	-	-	-	-	649.8	15	648.0	13
	491.6	481.5	-	-	506.8	65	485.0	155
$^{16}O^{109}AgF$	-	-	-	-	648.3	15	646.5	13
	491.6	481.5	-	-	506.8	65	485.0	155
$^{18}O^{107}AgF$	-	-	-	-	638.0	22	638.8	23
	472.9	463.3	-	-	490.0	56	467.0	138
$^{18}O^{109}AgF$	-	-	-	-	636.5	22	637.4	23
	472.9	463.3	-	-	490.0	56	467.0	138
$^{16}O^{107}AgF_2$	663.6	644.6	-	-	651.5	133	672.1	152
	-	-	-	-	555.9	5	576.1	6
	-	-	-	-	516.3	0	497.8	1
$^{16}O^{109}AgF_2$	662.3	643.0	-	-	650.0	133	670.5	152
	-	-	-	-	555.9	5	576.1	6
	-	-	-	-	515.7	0	497.2	1
$^{18}O^{107}AgF_2$	663.3	644.6	-	-	651.5	133	672.1	152
	-	-	-	-	555.8	5	576.0	6
	-	-	-	-	490.5	0	472.9	1
$^{18}O^{109}AgF_2$	661.9	643.0	-	-	650.0	133	670.5	152
	-	-	-	-	555.8	5	576.0	6
	-	-	-	-	489.9	0	472.3	1
$(F^{16}O^{107}AgF)$	834.6	840.0	-	-	975.7	303	956.8	230
	605.1	586.9	-	-	583.8	38	570.0	56
	-	-	-	-	429.0	23	300.0	114
$(F^{16}O^{109}AgF)$	834.6	840.0	-	-	975.7	303	956.8	230
	605.1	586.9	-	-	582.6	38	570.0	57
	-	-	-	-	429.0	23	299.5	114
$(F^{18}O^{107}AgF)$	811.0	815.2	-	-	946.1	283	928.5	213
	598.3	578.5	-	-	580.5	41	568.9	61
	-	-	-	-	409.9	19	285.1	101
$(F^{18}O^{109}AgF)$	811.0	815.2	-	-	946.1	284	928.5	213
	598.3	578.5	-	-	579.2	41	567.8	61
	-	-	-	-	409.8	19	285.0	101

[a] Frequencies in [cm^{-1}] and intensities in [km/mol]. [b] X. Wang, L. Andrews, F. Brosi, S. Riedel, *Chem. Eur. J.* **2013**, *19*, 1397. Values calculated at CCSD(T)/aug-cc-pVXZ level and B3LYP/aug-cc-pVTZ(-PP) level. **[c]** This work, values are calculated at B3LYP/aug-cc-pVTZ(-PP) or CCSD(T)/aug-cc-pVTZ(-PP) levels using the CFOUR program.

Supplementary Table 3 Experimental and calculated frequencies of copper fluorides and oxygen fluorides[a]

	Exp. (this work)		Exp. Ref.[b]		Calc.			
	(Ne)	(Ar)	(Ne)	(Ar)	B3LYP[c]	Int.	CCSD(T) [c]	Int.
Ne-^{63}CuF	625.3	-	-	-	628.0[b]	59	637.6[b]	-
Ne-^{65}CuF	623.4	-	-	-	625.6[b]	58	635.2[b]	-
Ar-^{63}CuF	-	615.4	-	615.9	631.2[b]	61	647.1[b]	-
Ar-^{65}CuF	-	612.8	-	610.6	628.8[b]	60	644.6[b]	-
$^{63}CuF_2$	766.2	743.4	766.1	743.6	786.9[b]	138	776.2[b]	-
$^{65}CuF_2$	761.8	739.2	761.9	739.0	782.3[b]	136	771.7[b]	-
$^{63}CuF_3$	-	-	786.1	760.9	782.1[b]	132	801.1[b]	-
$^{65}CuF_3$	-	-	781.8	759.4	777.5[b]	130	796.4[b]	-
$^{16}O^{63}CuF_2$	772.0	751.1	-	-	762.4	132	788.8	133
	-	-	-	-	621.8	5	641.5	4
	-	-	-	-	508.1	6	510.2	2
$^{16}O^{65}CuF_2$	767.7	746.5	-	-	758.0	131	784.2	132
	-	-	-	-	621.6	6	641.4	4
	-	-	-	-	506.7	6	509.1	2
$^{18}O^{63}CuF_2$	772.0	751.1	-	-	762.4	132	788.8	133
	-	-	-	-	621.3	6	641.4	4
	-	-	-	-	485.2	5	487.0	1
$^{18}O^{65}CuF_2$	767.7	746.5	-	-	758.0	131	784.2	132
	-	-	-	-	621.1	6	641.3	4
	-	-	-	-	483.7	5	485.5	1
($F^{16}O^{63}CuF$)	-	-	-	-	949.8	80	892.2	4
	736.0	715.3	-	-	744.2	131	761.7	197
	-	-	-	-	579.9	2	602.0	2
($F^{16}O^{65}CuF$)	-	-	-	-	949.6	81	891.5	5
	732.1	711.5	-	-	740.2	129	758.0	195
	-	-	-	-	579.9	2	601.9	2
($F^{18}O^{63}CuF$)	-	-	-	-	919.7	76	863.1	4
	728.5	707.7	-	-	737.5	130	754.7	190
	-	-	-	-	558.7	1	580.9	4
($F^{18}O^{65}CuF$)	-	-	-	-	919.6	77	862.4	5
	724.6	704.0	-	-	733.5	128	751.1	188
	-	-	-	-	558.5	1	580.7	4

[a] Frequencies in [cm^{-1}] and intensities in [km/mol]. [b] X. Wang, L. Andrews, F. Brosi, S. Riedel, *Chem. Eur. J.* **2013**, *19*, 1397. Values calculated at CCSD(T)/aug-cc-pVQZ level and B3LYP/aug-cc-pVTZ(-PP) level. [c] This work, values are calculated at the DFT-B3LYP/aug-cc-pVTZ(-PP) and the CCSD(T)/aug-cc-pVTZ(-PP) levels of theory using the CFOUR program.

Supplementary Table 4 Calculated thermochemistry of coinage metal oxide fluorides in kJ mol^{-1} [a]

Reaction	CCSD(T)/aVTZ(-PP)		B3LYP/aVTZ(-PP)	
Cu + OF → CuOF	−259.2	(−256.1)	−236.2	(−233.0)
Cu + OF → OCuF	−478.3	(−473.7)	−447.7	(−443.6)
Cu + OF_2 → FOCuF	−439.3	(−436.5)	−452.2	(−450.0)
Cu + OF_2 → $OCuF_2$	−464.3	(−462.8)	−447.0	(−446.6)
Ag + OF → AgOF	−192.5	(−190.1)	−171.7	(−169.2)
Ag + OF → OAgF	−221.9	(−219.5)	−243.5	(−241.7)
Ag + OF_2 → FOAgF	−217.2	(−217.7)	−253.8	(−253.8)
Ag + OF_2 → $OAgF_2$	−202.5	(−202.4)	−229.1	(−230.0)
Au + OF → AuOF	−166.7	(−163.6)	−153.8	(−150.5)
Au + OF → OAuF	−330.8	(−326.4)	−338.6	(−334.7)
Au + OF_2 → FOAuF	−280.8	(−277.3)	−305.7	(−303.8)
Au + OF_2 → $OAuF_2$	−292.2	(−291.2)	−300.3	(−300.2)

[a] Values in parentheses are corrected for ZPE from harmonic frequency analysis.

Supplementary Table 5 Calculated transition state energies at the UB3LYP-D3BJ/def2-TZVPP level for the monomolecular rearrangement from FOMF $^2A''$ to OMF_2 $^2B_2$

M		OMF_2	FMOF	TS(OMF_2 – FMOF)
Cu	Energy (au)	-1915.2709	-1915.2730	-1915.2626
	ΔE (kJ/mol)	6	0	22
Ag	Energy (au)	-421.7269	-421.7404	-421.7011
	ΔE (kJ/mol)	35	0	103
Au	Energy (au)	-410.4884	-410.4925	-410.4425
	ΔE (kJ/mol)	11	0	131

Supplementary Table 6 Orbital energies for OAuF $^3\Sigma^-$ ground state at equilibrium structure (for a corresponding MO diagram see Supplementary Scheme 1, for isovalue surface representations of MOs see Supplementary Figure 13)[a]

MO label	VDZ(-PP)	VTZ(-PP)	VQZ(-PP)
4 π	-0.3408[b]	-0.3301[b]	-0.3283[b]
9 σ	-0.4230	-0.4218	-0.4221
3 π	-0.5135	-0.5217	-0.5235
1 δ	-0.5296	-0.5324	-0.5336
8 σ	-0.5778	-0.5871	-0.5889
2 π	-0.6178	-0.6247	-0.6267
7 σ	-0.7598	-0.7585	-0.7588

[a] In hartree, calculated at sa-MCSCF level (orbitals optimized for $^3\Sigma^-$ state, with monitoring of the excited $^1\Delta$ and $^1\Sigma^+$ states) using the MOLPRO program. [b] MO-LCAO coefficients of largest magnitude for the antibonding 4 π orbital: |c(Au 3dπ)| = 0.3934, |c(O 2pπ)| = 0.4201, |c(F 2pπ)| = 0.0598 for VDZ(-PP); |c(Au 3dπ)| = 0.4101, |c(O 2pπ)| = 0.4950, |c(F 2pπ)| = 0.0488 for VTZ(-PP); |c(Au 3dπ)| = 0.4118, |c(O 2pπ)| = 0.4480, |c(F 2pπ)| = 0.0421 for VQZ(-PP).

Supplementary Table 7 List of species (including electronic ground state label and point group) for which quantum-chemical results are presented in Supplementary Dataset 1

Atoms	Diatomic Molecules	Triatomic Molecules	Tetratomic Molecules
O ^3P_g (K_h)<br>F ^2P_u (K_h)	OF $^2\Pi$ ($C_{\infty\mathrm{v}}$)	OF_2 $^1A_1$ ($C_{2\mathrm{v}}$)	
Au ^2S_g (K_h)	AuO $^2\Pi$ ($C_{\infty\mathrm{v}}$)<br>AuF $^1\Sigma^+$ ($C_{\infty\mathrm{v}}$)	AuOF $^1A'$ (C_s)<br>OAuF $^3\Sigma^-$ ($C_{\infty\mathrm{v}}$)	FAuOF $^2A''$ (C_s)<br>$OAuF_2$ $^2B_2$ ($C_{2\mathrm{v}}$)
Ag ^2S_g (K_h)	...	AgOF $^1A'$ (C_s)<br>OAgF $^3\Sigma^-$ ($C_{\infty\mathrm{v}}$)	FAgOF $^2A''$ (C_s)<br>$OAgF_2$ $^2B_2$ ($C_{2\mathrm{v}}$)
Cu ^2S_g (K_h)	...	CuOF $^1A'$ (C_s)<br>OCuF $^3\Sigma^-$ ($C_{\infty\mathrm{v}}$)	FCuOF $^2A''$ (C_s)<br>$OCuF_2$ $^2B_2$ ($C_{2\mathrm{v}}$)

Supplementary Note 1

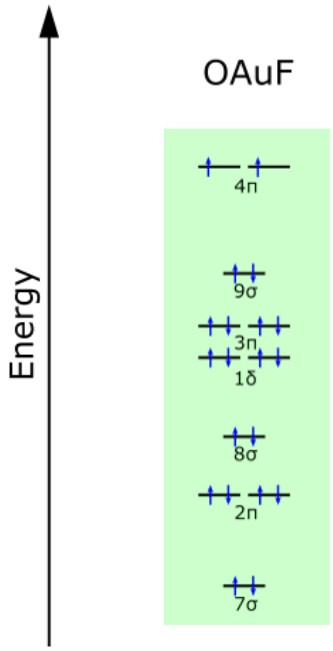

Molecular Orbitals and Spin Densities of OAuF

MO diagram for the molecular valence orbitals of OAuF $^3\Sigma^-$ (cf. also Supplementary Table 6 for quantitative data from sa-MCSCF calculations and Supplementary Figure 13 for isovalue surface representations).

The relative energies of the molecular orbitals of OAuF were obtained at the Kohn-Sham DFT level, using the B3LYP hybrid functional. Qualitatively these energies do not differ much from those obtained with various basis sets at the sa-MCSCF level (where all space- and spin-symmetry requirements are met). The σ-bond in OAuF can be formulated as a three-centre-four-electron bond involving the pσ orbitals of the terminal atoms and a 5dσ-6s hybrid orbital of gold. The singly occupied 4π molecular orbitals are predominantly of antibonding π* O-Au character (see Supplementary Figure 12). Thus, the interactions of the O, F p_z orbitals with Au 6s and $5d_{z^2}$ yield one strongly bonding 7σ, two essentially nonbonding 8σ (mainly of O/F p character) and 9σ (mainly of Au 5d character), and the virtual antibonding 10σ (mainly of Au 6s character) MO's. The Au ($5d_{xz}$, $5d_{yz}$) orbitals are involved in the π-bonding 2π, a nonbonding 3π (primarily of F ($2p_x$, $2p_y$) character, weakly Au-O π bonding and weakly Au-F π antibonding), and the strongly Au-O π* antibonding 4π MO's. The 1δ MO's consists of the purely nonbonding Au $5d_{xy}$, $5d_{x^2-y^2}$ orbitals.

Supplementary Methods

Experimental and quantum-chemical details

The experimental set-up used for the laser ablation of coinage metals (Au, Ag, Cu) and their reaction with OF_2 in different host gases, as well as their deposition at 5-15 K using a closed-cycle helium cryostat (Sumitomo Heavy Industries, RDK-205D) inside a vacuum chamber has been described in more detail in our previous works.[1] The laser beam was focused using a plano convex lens with a diameter of 25.4 mm and a focal distance of 125.0 mm. $^{16/18}OF_2$ was synthesized by a known procedure using elemental fluorine and $^{16/18}OH_2$ dispersed in solid NaF.[2] The Nd:YAG laser fundamental (Continuum, Minilite II, 1064 nm, 10 Hz repetition rate with 7 ns pulse width) with a pulse energy of up to 50 mJ/cm^2 was focused onto the metal targets, which gave an energetic plasma beam reacting with OF_2 and spreading toward the cold rhodium-plated mirror. FTIR spectra were recorded on a Bruker Vertex 80v spectrometer at a 0.5 cm^{-1} resolution and with a 0.5 cm^{-1} accuracy using an MCTB detector. Matrix samples were annealed at different temperatures, and selected samples were subjected to irradiation by a medium pressure mercury arc street lamp with the globe removed ($\lambda > 220$ nm). Selective irradiations with λ = 590, 528, 455-405 or 365 nm and 266 nm were carried out using LED's or a solid-state Nd:YAG laser source with quadrupled frequency, respectively.

Quantum-chemical details: Molecular structures of all species in their electronic ground states were optimized at the CCSD(T) level, based on space- and spin-symmetry adapted configuration state functions (CSFs) as reference wavefunctions (closed-shell states and different types of open-shell states occurred, see Supplementary Table 7). Correlation consistent triple-ζ all-electron basis sets (aug-cc-pVTZ)[3] were used for atoms of oxygen and fluorine, scalar-relativistic energy-consistent effective core potentials (19-valence-electron ECPs)[4] and suitably chosen corresponding valence basis sets (aug-cc-pVTZ-PP)[5] were used for atoms of the coinage metals. The required symmetry-defect-free spatial orbitals and CSFs were provided by restricted (open-shell) Hartree-Fock (R(O)HF) calculations or state-averaged multi-configuration self-consistent field (sa-MCSCF) calculations in a minimal space of active orbitals. These calculations were carried out with either the MOLPRO program[6] or the CFOUR program.[7] Restricted CCSD(T) calculations, performed for open-shell states with MOLPRO, are free from spin contamination, i.e., the expectation value of S^2-$S_z(S_z$+1) vanishes exactly. In addition, the GAUSSIAN program[8] was used to perform calculations at the unrestricted Kohn-Sham DFT level, using the B3LYP hybrid functional.[9] Minima on potential energy surfaces were characterized by normal mode analysis. Thermochemical data is provided without counterpoise correction, but includes zero-point energy corrections as obtained from harmonic vibrational frequencies. Within the study of the gold-containing species, smaller basis sets were also employed (cc-pVDZ, aug-cc-pVDZ, cc-pVTZ).[3, 5] Calculations for the compound OAuF were performed even with larger basis sets (cc-pVQZ, aug-cc-pVQZ).[3, 5] Anharmonic vibrational frequencies were calculated for all di- and triatomic gold-containing species included in this study, via Dunham analysis[10] or by use of a vibrational self-consistent field procedure (VSCF)[11].

Supplementary References

[1] T. Schlöder, T. Vent-Schmidt, S. Riedel, *Angew. Chem., Int. Ed.* **2012**, *51*, 12063-12067.

[2] A. H. Borning, K. E. Pullen, *Inorg. Chem.* **1969**, *8*, 1791.

[3] a) T. H. Dunning, Jr., *J. Chem. Phys.* **1989**, *90*, 1007-1023; b) R. A. Kendall, T. H. Dunning, Jr., R. J. Harrison, *J. Chem. Phys.* **1992**, *96*, 6796-6806.

[4] D. Figgen, G. Rauhut, M. Dolg, H. Stoll, *Chem. Phys.* **2005**, *311*, 227-244.

[5] K. A. Peterson, C. Puzzarini, *Theor. Chem. Acc.* **2005**, *114*, 283-296.

[6] a) H.-J. Werner, P. J. Knowles, G. Knizia, F. R. Manby, M. Schütz, *Wiley Interdisciplinary Reviews: Computational Molecular Science* **2012**, *2*, 242-253; b) MOLPRO, version 2015.1, a package of ab initio programs, H.-J. Werner, P. J. Knowles, G. Knizia, F. R. Manby, M. Schütz, P. Celani, W. Györffy, D. Kats, T. Korona, R. Lindh, A. Mitrushchenkov, G. Rauhut, K. R. Shamasundar, T. B. Adler, R. D. Amos, A. Bernhardsson, A. Berning, D. L. Cooper, M. J. O. Deegan, A. J. Dobbyn, F. Eckert, E. Goll, C. Hampel, A. Hesselmann, G. Hetzer, T. Hrenar, G. Jansen, C. Köppl, Y. Liu, A. W. Lloyd, R. A. Mata, A. J. May, S. J. McNicholas, W. Meyer, M. E. Mura, A. Nicklass, D. P. O'Neill, P. Palmieri, D. Peng, K. Pflüger, R. Pitzer, M. Reiher, T. Shiozaki, H. Stoll, A. J. Stone, R. Tarroni, T. Thorsteinsson, M. Wang, **2015**, see http://www.molpro.net.

[7] CFOUR, a quantum-chemical program package written by J. F. Stanton, J. Gauss, L. Cheng, M. E. Harding, D. A. Matthews, P. G. Szalay with contributions from A. A. Auer, R. J. Bartlett, U. Benedikt, C. Berger, D. E. Bernholdt, Y. J. Bomble, L. Cheng, O. Christiansen, F. Engel, R. Faber, M. Heckert, O. Heun, C. Huber, T.-C. Jagau, D. Jonsson, J. Jusélius, K. Klein, W. J. Lauderdale, F. Lipparini, D. A. Matthews, T. Metzroth, L. A. Mück, D. P. O'Neill, D. R. Price, E. Prochnow, C. Puzzarini, K. Ruud, F. Schiffmann, W. Schwalbach, C. Simmons, S. Stopkowicz, A. Tajti, J. Vázquez, F. Wang, J. D. Watts and the integral packages MOLECULE (J. Almlöf and P. R. Taylor), PROPS (P. R. Taylor), ABACUS (T. Helgaker, H. J. Aa. Jensen, P. Jørgensen, and J. Olsen), and ECP routines by A. V. Mitin and C. van Wüllen. For the current version, see http://www.cfour.de.

[8] M. J. Frisch, G. W. Trucks, H. B. Schlegel, G. E. Scuseria, M. A. Robb, J. R. Cheeseman, G. Scalmani, V. Barone, B. Mennucci, G. A. Petersson, H. Nakatsuji, M. Caricato, X. Li, H. P. Hratchian, A. F. Izmaylov, J. Bloino, G. Zheng, J. L. Sonnenberg, M. Hada, M. Ehara, K. Toyota, R. Fukuda, J. Hasegawa, M. Ishida, T. Nakajima, Y. Honda, O. Kitao, H. Nakai, T. Vreven, J. A. Montgomery Jr., J. E. Peralta, F. Ogliaro, M. Bearpark, J. J. Heyd, E. Brothers, K. N. Kudin, V. N. Staroverov, R. Kobayashi, J. Normand, K. Raghavachari, A. Rendell, J. C. Burant, S. S. Iyengar, J. Tomasi, M. Cossi, N. Rega, N. J. Millam, M. Klene, J. E. Knox, J. B. Cross, V. Bakken, C. Adamo, J. Jaramillo, R. Gomperts, R. E. Stratmann, O. Yazyev, A. J. Austin, R. Cammi, C. Pomelli, J. W. Ochterski, R. L. Martin, K. Morokuma, V. G. Zakrzewski, G. A. Voth, P. Salvador, J. J. Dannenberg, S. Dapprich, A. D. Daniels, Ö. Farkas, J. B. Foresman, J. V. Ortiz, J. Cioslowski and D. J. Fox, Gaussian 09, Revision B.01, Gaussian, Inc., Wallingford CT, **2009**.

[9] a) A. D. Becke, *J. Chem. Phys.* **1993**, *98*, 5648-5652; b) C. Lee, W. Yang, R. G. Parr, *Physical Review B* **1988**, *37*, 785-789; c) B. Miehlich, A. Savin, H. Stoll, H. Preuss, *Chem. Phys. Lett.* **1989**, *157*, 200-206.

[10] J. L. Dunham, *Phys. Rev.* **1932**, *41*, 721-731.

[11] a) G. Rauhut, *J. Chem. Phys.* **2004**, *121*, 9313-9322; b) T. Hrenar, H.-J. Werner, G. Rauhut, *J. Chem. Phys.* **2007**, *126*, 134108/1-9; c) G. Rauhut, T. Hrenar, *Chem. Phys.* **2008**, *346*, 160-166.

[12] K. P. Huber, G. Herzberg, Constants of Diatomic Molecules (Molecular Spectra and Molecular Structure, Vol. IV), Van Nostrand Reinhold, New York, **1979**.

[13] T. Shimanouchi, Tables of Molecular Vibrational Frequencies --- Consolidated Volume I, Nat. Stand. Ref. Data Ser., Nat. Bur. Stand., *39*, **1972**.
