# Supplementary material for: Oxygen radical character in group 11 oxygen fluorides
Source: Nat Commun. 2018 Mar 28;9:1267. doi: 10.1038/s41467-018-03630-0 (PMC5871800; doi:10.1038/s41467-018-03630-0)
Supplement: Supplementary file 2 — Description of Additional Supplementary Files(PDF 167 kb) [file 41467_2018_3630_MOESM2_ESM.pdf]

## Description of Additional Supplementary Files

**File Name: Supplementary Data 1**
**Description:** contains all quantum-chemical results such as energies, electronic configurations, and frequencies etc. which have been computed during the investigation of group 11 oxygen fluorides.
