# Supplementary material for: Oxygen radical character in group 11 oxygen fluorides
Source: Nat Commun. 2018 Mar 28;9:1267. doi: 10.1038/s41467-018-03630-0 (PMC5871800; doi:10.1038/s41467-018-03630-0)
Supplement: Supplementary file 3 — Supplementary Data 1 (PDF 336 kb) [file 41467_2018_3630_MOESM3_ESM.pdf]

Supplementary Dataset 1

List of species (including electronic ground state label and point group) for which quantum-chemical results are presented in this Supplementary Dataset 1

Atoms	Diatomic Molecules	Triatomic Molecules	Tetratomic Molecules
O ^3P_g (K_h)	OF $^2\Pi$ ($C_{\infty\mathrm{v}}$)	OF_2 $^1A_1$ ($C_{2\mathrm{v}}$)	—
F ^2P_u (K_h)	—	—	—
Au ^2S_g (K_h)	AuO $^2\Pi$ ($C_{\infty\mathrm{v}}$)	AuOF $^1A'$ (C_s)	FAuOF $^2A''$ (C_s)
	AuF $^1\Sigma^+$ ($C_{\infty\mathrm{v}}$)	OAuF $^3\Sigma^-$ ($C_{\infty\mathrm{v}}$)	$OAuF_2$ $^2B_2$ ($C_{2\mathrm{v}}$)
Ag ^2S_g (K_h)	—	AgOF $^1A'$ (C_s)	FAgOF $^2A''$ (C_s)
	—	OAgF $^3\Sigma^-$ ($C_{\infty\mathrm{v}}$)	$OAgF_2$ $^2B_2$ ($C_{2\mathrm{v}}$)
Cu ^2S_g (K_h)	—	CuOF $^1A'$ (C_s)	FCuOF $^2A''$ (C_s)
	—	OCuF $^3\Sigma^-$ ($C_{\infty\mathrm{v}}$)	$OCuF_2$ $^2B_2$ ($C_{2\mathrm{v}}$)

Note: See list of Supplementary References for references cited in this Supplementary Dataset 1

O — F

Results for O X 3-P even parity [3-(B1g & B2g & B3g)]
===
Open-shell system, 8 electrons
Electron configuration: (1s)2 (2s)2 (2p)4 [2(b1u & b2u & b3u)4]

Total energies from RHF (minimal sa-MCSCF in D2h), from MRCI and from RCCSD(T):

BASIS	EHF/AU	EMRCI/AU	ECC/AU	T1(*)
VDZ	-74.78618804	-74.90703660	-74.90983830	0.0105
AVDZ	-74.78905189	-74.92088442	-74.92547597	0.0197
VTZ	-74.80307839	-74.96746864	-74.97370484	0.0143
AVTZ	-74.80359970	-74.97157369	-74.97866419	0.0186
VQZ	-74.80797536	-74.98583428	-74.99326555	0.0161
AVQZ	-74.80807838	-74.98721229	-74.99495450	0.0181

(*) T1 diagnostic from RCCSD(T)

Total energies from KS-DFT (B3LYP) [GAUSSIAN], and from RCCSD(T) [CFOUR]:

BASIS	EDFT/AU	ECC/AU
VDZ	-75.06849692	-74.90989194
AVDZ	-75.07716215	-74.92559617
VTZ	-75.09186254	-74.97386348
AVTZ	-75.09417782	-74.97885723

Results for F X 2-P odd parity [2-(B1u & B2u & B3u)]
==
Open-shell system, 9 electrons
Electron configuration: (1s)2 (2s)2 (2p)5 [2(b1u & b2u & b3u)5]

Total energies from RHF (minimal sa-MCSCF in D2h), from MRCI and from RCCSD(T):

BASIS	EHF/AU	EMRCI/AU	ECC/AU	T1(*)
VDZ	-99.37108022	-99.52349520	-99.52751764	0.0067
AVDZ	-99.37595738	-99.54367627	-99.54997163	0.0140
VTZ	-99.39919372	-99.61170334	-99.62022622	0.0096
AVTZ	-99.40010315	-99.61798722	-99.62767440	0.0129
VQZ	-99.40697999	-99.63989237	-99.65009123	0.0109
AVQZ	-99.40715895	-99.64209917	-99.65273285	0.0124

(*) T1 diagnostic from RCCSD(T)

Total energies from KS-DFT (B3LYP) [GAUSSIAN], and from RCCSD(T) [CFOUR]:

BASIS	EDFT/AU	ECC/AU
VDZ	-99.72660073	-99.52754248
AVDZ	-99.73949531	-99.55004116
VTZ	-99.76286603	-99.62030949
AVTZ	-99.76614056	-99.62777999

Quantum-Chemical Results for the Atoms Cu, Ag and Au

Cu — Ag — Au

```
Results for Cu X 2-S even parity
================================
Open-shell system, Cu ECP10MDF, 19 electrons
Electron configuration: (3s)2 (3p)6 (3d)10 (4s)1

Total energies from KS-DFT (B3LYP) [GAUSSIAN], and from RCCSD(T) [CFOUR]:

BASIS           EDFT/AU                              ECC/AU
VDZ-PP     -197.30218361                        -196.53510112
AVDZ-PP    -197.31182995                        -196.58426464
VTZ-PP     -197.32368315                        -196.63932923
AVTZ-PP    -197.32497035                        -196.65998983



Results for Ag X 2-S even parity
================================
Open-shell system, Ag ECP28MDF, 19 electrons
Electron configuration: (4s)2 (4p)6 (4d)10 (5s)1

Total energies from KS-DFT (B3LYP) [GAUSSIAN], and from RCCSD(T) [CFOUR]:

BASIS           EDFT/AU                              ECC/AU
VDZ-PP     -146.97047714                        -146.33568405
AVDZ-PP    -146.97132457                        -146.37609174
VTZ-PP     -146.97302555                        -146.43605702
AVTZ-PP    -146.97313989                        -146.45225125



Results for Au X 2-S even parity
================================
Open-shell system, Au ECP60MDF, 19 electrons
Electron configuration: (5s)2 (5p)6 (5d)10 (6s)1

Total energies from RHF and from RCCSD(T):

BASIS           EHF/AU                               ECC/AU        T1(*)
VDZ-PP     -134.77960928                        -135.06357301    0.0075
AVDZ-PP    -134.77979159                        -135.09121493    0.0135
VTZ-PP     -134.78077946                        -135.14859900    0.0124
AVTZ-PP    -134.78079703                        -135.16016252    0.0141
VQZ-PP     -134.78085444                        -135.18302313    0.0139
AVQZ-PP    -134.78085838                        -135.18807530    0.0148

(*) T1 diagnostic from RCCSD(T)

Total energies from KS-DFT (B3LYP) [GAUSSIAN], and from RCCSD(T) [CFOUR]:

BASIS           EDFT/AU                              ECC/AU
VDZ-PP     -135.74831345                        -135.06368569
AVDZ-PP    -135.74860106                        -135.09134597
VTZ-PP     -135.75012836                        -135.14872129
AVTZ-PP    -135.75015933                        -135.16029175
```

Quantum-Chemical Results for the Molecule OF

O—F

Results for O-F X 2-Pi [2-(B1 & B2)]
====================================
Open-shell system, 17 electrons
Electron configuration:
- closed-shell part: sigma 1-5, pi 1 [a1 1-5, b1 1, b2 1]
- open-shell part: (2pi)3 [2(b1 & b2)3]

Type and number of normal modes: Sigma (A) 1 [A1 1]

Optimized bond length, associated total energy, dipole moment:

BASIS	METHOD	O-F/ANG	E/AU	MU/D
AVTZ	B3LYP [GAUSSIAN]	1.35075	-174.94864341	0.0213
AVTZ	CCSD(T) [CFOUR]	1.36012	-174.68503850	--

Normal mode analysis (harmonic frequency, absolute IR intensity in km/mol):

Vibration Nr	WE*CM [160]	[180]		WE*CM [160]	[180]
>> B3LYP [GAUSSIAN]:					
				>> AVTZ:	
1			A1	1115.43 (46.95)	1081.19 (44.11)
ZPE				557.71	540.59
>> CCSD(T) [CFOUR]:					
				>> AVTZ:	
1			A1	1054.17 (34.45)	1021.81 (34.24)
ZPE				527.09	510.90

Spectroscopic constants (from polynomials through lowest 7 points of PECs):

			[160]			[180]		
BASIS	RE/ANG	E(RE)/AU	WE*CM	WEXE*CM	NU01*CM (+)	WE*CM	WEXE*CM	NU01*CM (+)
>> RHF (minimal sa-MCSCF in C2v):								
VDZ	1.3223	-174.1292356	1181.28	5.62	1170.04	1145.01	5.28	1134.45
AVDZ	1.3185	-174.1431693	1214.65	6.05	1202.55	1177.36	5.68	1166.00
VTZ	1.3130	-174.1859207	1244.83	6.06	1232.71	1206.62	5.69	1195.24
AVTZ	1.3132	-174.1885352	1246.12	6.05	1234.02	1207.86	5.68	1196.50
>> MRCI:								
VDZ	1.3526	-174.4662077	1061.89	7.54	1046.81	1029.29	7.09	1015.11
AVDZ	1.3528	-174.5025293	1081.09	6.99	1067.11	1047.90	6.57	1034.76
VTZ	1.3334	-174.6208871	1153.01	7.20	1138.61	1117.61	6.77	1104.07
AVTZ	1.3345	-174.6323187	1147.97	7.01	1133.95	1112.73	6.58	1099.57
>> RCCSD(T):								
VDZ	1.3749	-174.4980034	977.41	11.45	954.51	947.40	10.76	925.88
AVDZ	1.3780	-174.5421920	991.17	10.03	971.11	960.74	9.42	941.90
VTZ	1.3568	-174.6696032	1065.18	10.14	1044.90	1032.48	9.52	1013.44
AVTZ	1.3598	-174.6844695	1054.17	10.03	1034.11	1021.81	9.42	1002.97

(+) NU01 = WE - 2*WEXE

Ref.: ^{16}OF X$^2\Pi$, $r_e = 1.326$ Å (calcd. HF-SCF), $\omega_e = 1028.7$ cm^{-1} (IR and Raman spectra in Ar matrix) [12]

Quantum-Chemical Results for the Molecule AuO

Au—O

Results for Au-O X 2-Pi [2-(B1 & B2)]
=====================================

Open-shell system, Au ECP60MDF, 27 electrons
Electron configuration:
- closed-shell part: sigma 1-6, pi 2, delta 1 [a1 1-7, a2 1, b1 1-2, b2 1-2]
- open-shell part: (3pi)3 [3(b1 & b2)3]

Type and number of normal modes: Sigma (A) 1 [A1 1]

Spectroscopic constants (from polynomials through lowest 7 points of PECs):

BASIS	RE/ANG	E(RE)/AU	[160] WE*CM	[160] WEXE*CM	[160] NU01*CM (+)	[180] WE*CM	[180] WEXE*CM	[180] NU01*CM (+)
>> RHF (minimal sa-MCSCF in C2v):								
VDZ(-PP)	1.9908	-209.5580129	547.68	2.51	542.66	518.71	2.25	514.21
AVDZ(-PP)	2.0009	-209.5671650	534.47	2.35	529.77	506.20	2.11	501.98
VTZ(-PP)	1.9835	-209.5815665	545.81	2.41	540.99	516.94	2.16	512.62
AVTZ(-PP)	1.9811	-209.5842900	542.35	2.51	537.33	513.67	2.26	509.15
>> MRCI:								
VDZ(-PP)	1.9360	-209.9710718	566.45	2.85	560.75	536.49	2.55	531.39
AVDZ(-PP)	1.9495	-210.0068929	549.57	2.30	544.97	520.50	2.06	516.38
VTZ(-PP)	1.9245	-210.0995743	572.19	2.53	567.13	541.92	2.27	537.38
AVTZ(-PP)	1.9247	-210.1139112	570.96	2.66	565.64	540.76	2.38	536.00
>> RCCSD(T):								
VDZ(-PP)	1.9145	-210.0323923	549.83	7.69	534.45	520.75	6.90	506.95
AVDZ(-PP)	1.9288	-210.0804566	531.07	2.59	525.89	502.98	2.33	498.32
VTZ(-PP)	1.8958	-210.1900364	565.82	3.46	558.90	535.89	3.10	529.69
AVTZ(-PP)	1.8977	-210.2103268	564.99	3.50	557.99	535.11	3.14	528.83

(+) NU01 = WE - 2*WEXE

Quantum-Chemical Results for the Molecule AuF

Au—F

Results for Au-F X 1-Sigma+ [1-A1]
==================================

Closed-shell system, Au ECP60MDF, 28 electrons
Electron configuration: sigma 1-6, pi 1-3, delta 1 [a1 1-7, a2 1, b1 1-3, b2 1-3]
Type and number of normal modes: Sigma (A) 1 [A1 1]

Optimized bond length, associated total energy, dipole moment, and results from normal mode analysis (harmonic oscillator approximation):

BASIS	METHOD	AU-F/ANG	E/AU	T1(+)	MU/D	WE*CM	ZPE*CM
VDZ(-PP)	RHF	1.97689	-234.18902682	--	5.7696	542.62	271.31
VDZ(-PP)	MP2	1.92614	-234.68446217	--	4.2794	577.78	288.89
VDZ(-PP)	CCSD(T)	1.94748	-234.68233465	0.0359	4.3283 (*)	550.40	275.20
AVDZ(-PP)	RHF	1.99187	-234.20416341	--	5.9502	520.22	260.11
AVDZ(-PP)	MP2	1.94673	-234.74584829	--	4.7222	552.63	276.32
AVDZ(-PP)	CCSD(T)	1.96643	-234.74297058	0.0362	4.7516 (*)	532.62	266.31
VTZ(-PP)	RHF	1.97262	-234.22570175	--	5.7474	530.49	265.24
VTZ(-PP)	MP2	1.91482	-234.87557388	--	4.3566	576.47	288.24
VTZ(-PP)	CCSD(T)	1.93645	-234.86958875	0.0320	4.4814 (*)	551.71	275.86
AVTZ(-PP)	RHF	1.97495	-234.22994006	--	5.7471	519.00	259.50
AVTZ(-PP)	MP2	1.92149	-234.90041750	--	4.4608	563.36	281.68
AVTZ(-PP)	CCSD(T)	1.94202	-234.89453116	0.0309	4.5843 (*)	541.41	270.70

(+) T1 diagnostic from CCSD(T)
(*) CCSD dipole moment (nonrelaxed)

Spectroscopic constants (from polynomials through lowest 7 points of PECs):

BASIS	RE/ANG	E(RE)/AU	WE*CM	WEXE*CM	NU01*CM (+)
>> RHF:					
VDZ(-PP)	1.9769	-234.1890268	542.60	2.27	538.06
AVDZ(-PP)	1.9919	-234.2041634	520.20	2.52	515.16
VTZ(-PP)	1.9726	-234.2257017	530.46	2.38	525.70
AVTZ(-PP)	1.9749	-234.2299401	518.97	2.47	514.03
>> MP2:					
VDZ(-PP)	1.9261	-234.6844622	577.77	2.42	572.93
AVDZ(-PP)	1.9467	-234.7458483	552.60	2.49	547.62
VTZ(-PP)	1.9148	-234.8755739	576.46	2.49	571.48
AVTZ(-PP)	1.9215	-234.9004175	563.34	2.59	558.16
>> CCSD(T):					
VDZ(-PP)	1.9475	-234.6823347	550.41	2.90	544.61
AVDZ(-PP)	1.9664	-234.7429706	532.62	2.79	527.04
VTZ(-PP)	1.9364	-234.8695887	551.72	2.82	546.08
AVTZ(-PP)	1.9420	-234.8945312	541.39	2.81	535.77

(+) NU01 = WE - 2*WEXE

Quantum-Chemical Results for the Molecule Au_2

Au—Au

```
Results for Au2 X 1-Sigmag+ [1-Ag]
==================================
Closed-shell system, Au ECP60MDF, 38 electrons
Electron configuration:
sigmag 1-4, sigmau 1-3, pig 1-2, piu 1-2, deltag 1, deltau 1
[ag 1-5, b1g 1, b2g 1-2, b3g 1-2, au 1, b1u 1-4, b2u 1-2, b3u 1-2]
Type and number of normal modes: Sigmag (Ag) 1 [Ag 1]
```

Optimized bond length, associated total energy, and results from normal mode analysis (harmonic oscillator approximation):

BASIS	METHOD	AU-AU/ANG	E/AU	T1(+)	WE*CM	ZPE*CM
VDZ-PP	RHF	2.60914	-269.58869725	--	158.05	79.02
VDZ-PP	MP2	2.47980	-270.22982968	--	190.77	95.39
VDZ-PP	CCSD(T)	2.52531	-270.20039828	0.0169	175.53	87.76
AVDZ-PP	RHF	2.60720	-269.59019269	--	156.76	78.38
AVDZ-PP	MP2	2.47281	-270.29384918	--	197.43	98.72
AVDZ-PP	CCSD(T)	2.52037	-270.25824200	0.0208	180.14	90.07
VTZ-PP	RHF	2.60682	-269.59172127	--	156.26	78.13
VTZ-PP	MP2	2.46002	-270.41544610	--	199.03	99.51
VTZ-PP	CCSD(T)	2.50932	-270.37286502	0.0184	181.54	90.77
AVTZ-PP	RHF	2.60067	-269.59279220	--	156.69	78.34
AVTZ-PP	MP2	2.45075	-270.44251882	--	201.88	100.94
AVTZ-PP	CCSD(T)	2.50021	-270.39952024	0.0191	184.07	92.03

(+) T1 diagnostic from CCSD(T)

Spectroscopic constants (from polynomials through lowest 7 points of PECs):

BASIS	RE/ANG	E(RE)/AU	WE*CM	WEXE*CM	NU01*CM (+)
>> RHF:					
VDZ-PP	2.6091	-269.5886973	158.04	0.33	157.38
AVDZ-PP	2.6072	-269.5901927	156.76	0.33	156.10
VTZ-PP	2.6068	-269.5917213	156.25	0.33	155.59
AVTZ-PP	2.6007	-269.5927922	156.68	0.33	156.02
>> MP2:					
VDZ-PP	2.4798	-270.2298297	190.77	0.38	190.01
AVDZ-PP	2.4728	-270.2938492	197.42	0.39	196.64
VTZ-PP	2.4600	-270.4154461	199.02	0.40	198.22
AVTZ-PP	2.4508	-270.4425188	201.87	0.40	201.07
>> CCSD(T):					
VDZ-PP	2.5253	-270.2003983	175.53	0.40	174.73
AVDZ-PP	2.5203	-270.2582420	180.16	0.42	179.32
VTZ-PP	2.5093	-270.3728650	181.56	0.42	180.72
AVTZ-PP	2.5002	-270.3995202	184.08	0.42	183.24

(+) NU01 = WE - 2*WEXE

Quantum-Chemical Results for the Molecule OF_2

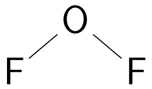

Results for F-O-F X 1-A1
========================

Closed-shell system, 26 electrons
Electron configuration: a1 1-6, a2 1, b1 1-2, b2 1-4
Type and number of normal modes: A1 1-2, B2 1

Optimized structural parameters, associated total energy, and dipole moment:

BASIS	METHOD	O-F/ANG	F-O-F/DEG	E/AU	T1	MU/D	COMMENT
VDZ	RHF	1.3469	103.326	-273.46882146	--	0.3474	
VDZ	MP2	1.4193	103.065	-274.04524785	--	0.3184	
VDZ	CCSD(T)	1.4357	103.016	-274.07251885	0.0152	--	
AVDZ	RHF	1.3401	103.121	-273.49451365	--	0.3229	
AVDZ	MP2	1.4220	102.591	-274.11548451	--	0.1418	
AVDZ	CCSD(T)	1.4325	102.682	-274.14671514	0.0185	--	
VTZ	RHF	1.3354	103.532	-273.56590292	--	0.3062	
VTZ	MP2	1.4009	103.141	-274.31713517	--	0.2854	
VTZ	CCSD(T)	1.4097	103.164	-274.34928380	0.0150	--	
AVTZ	RHF	1.3349	103.493	-273.57087701	--	0.3084	
AVTZ	B3LYP	1.40284	103.922	-274.77582157	--	0.3244	[GAUSSIAN]
AVTZ	MP2	1.4034	102.919	-274.34108691	--	0.1253	
AVTZ	CCSD(T)	1.4119	102.983	-274.37511552	0.0167	--	
AVTZ	CCSD(T)	1.41183	102.76	-274.37511553	--	--	[CFOUR]

Normal mode analysis (harmonic frequencies and relative IR intensities):

Vibration Nr		WE*CM [160]	[180]		WE*CM [160]	[180]
>> RHF:						
		>> VDZ:			>> AVDZ:	
1	A1	577.56 (3.01)	572.39 (3.36)	A1	588.75 (1.82)	583.40 (2.04)
2	B2	1136.46 (100.00)	1098.25 (100.00)	B2	1176.81 (100.00)	1137.29 (100.00)
3	A1	1155.17 (19.67)	1119.01 (19.09)	A1	1190.15 (15.97)	1153.03 (15.58)
ZPE		1434.60	1394.82		1477.86	1436.86
		>> VTZ:			>> AVTZ:	
1	A1	593.36 (2.11)	587.90 (2.36)	A1	594.31 (1.99)	588.82 (2.23)
2	B2	1209.17 (100.00)	1168.46 (100.00)	B2	1211.14 (100.00)	1170.38 (100.00)
3	A1	1215.25 (17.71)	1177.49 (17.27)	A1	1216.32 (16.18)	1178.57 (15.77)
ZPE		1508.89	1466.93		1510.88	1468.88

Ref.: $^{16}OF_2$ gas phase IR spectrum: ν_1 928/cm (A_1, sym str, strong), ν_2 461/cm (A_1, bend, very strong), ν_3 831/cm (B_2, antisym str, strong) [13]

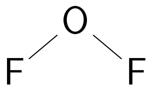

```
Results for F-O-F X 1-A1 (continued)
========================
Closed-shell system, 26 electrons
Electron configuration: a1 1-6, a2 1, b1 1-2, b2 1-4
Type and number of normal modes: A1 1-2, B2 1

Normal mode analysis (harmonic frequencies and relative IR intensities, continued):

    Vibration       WE*CM                                        WE*CM
       Nr           [16O]              [18O]                     [16O]              [18O]

>> B3LYP [GAUSSIAN] (absolute IR intensities in km/mol):
                                                              >> AVTZ:
       1                                                  A1   481.66 (  0.64)    477.43 (  0.68)
       2                                                  B2   893.89 (106.80)    863.73 ( 99.55)
       3                                                  A1  1018.83 ( 10.58)    986.77 (  9.71)
     ZPE                                                      1197.19            1163.97

>> MP2:
                 >> VDZ:                                      >> AVDZ:
       1      A1   464.35 (  1.60)    460.39 (  1.82)    A1   460.21 (  0.49)    456.34 (  0.58)
       2      B2   848.50 (100.00)    820.01 (100.00)    B2   844.72 (100.00)    816.44 (100.00)
       3      A1   923.73 ( 19.75)    894.41 ( 19.35)    A1   926.30 ( 13.48)    896.80 ( 13.32)
     ZPE          1118.29            1087.41                  1115.61            1084.79

                 >> VTZ:                                      >> AVTZ:
       1      A1   485.69 (  0.93)    481.48 (  1.07)    A1   481.64 (  0.63)    477.49 (  0.73)
       2      B2   916.57 (100.00)    885.79 (100.00)    B2   900.98 (100.00)    870.75 (100.00)
       3      A1   985.44 ( 15.54)    954.31 ( 15.29)    A1   974.54 ( 12.93)    943.70 ( 12.74)
     ZPE          1193.85            1160.79                  1178.58            1145.98

>> CCSD(T):
                 >> VDZ:                                      >> AVDZ:
       1      A1   437.01             433.63             A1   441.04             437.56
       2      B2   758.52             733.07             B2   791.22             764.71
       3      A1   857.31             829.43             A1   889.30             860.54
     ZPE          1026.42             998.07                  1060.78            1031.40

                 >> VTZ:                                      >> AVTZ:
       1      A1   469.19             465.31             A1   466.14             462.30
       2      B2   867.35             838.22             B2   859.39             830.56
       3      A1   950.06             919.67             A1   945.06             914.82
     ZPE          1143.30            1111.60                  1135.30            1103.84

>> CCSD(T) [CFOUR] (absolute IR intensities in km/mol):
                                                              >> AVTZ:
       1                                                  A1   466.25 (  0.51)    462.41 (  0.54)
       2                                                  B2   859.46 ( 63.74)    830.62 ( 59.54)
       1                                                  A1   945.11 (  7.52)    914.87 (  6.88)
     ZPE                                                      1135.41            1103.95
```

Ref.: $^{16}OF_2$ gas phase IR spectrum: ν_1 928/cm (A_1, sym str, strong), ν_2 461/cm (A_1, bend, very strong), ν_3 831/cm (B_2, antisym str, strong) [13]

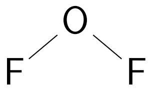

```
Results for F-O-F X 1-A1 (continued)
========================
Closed-shell system, 26 electrons
Electron configuration: a1 1-6, a2 1, b1 1-2, b2 1-4
Type and number of normal modes: A1 1-2, B2 1

Results from VSCF calculation on CCSD(T) PES:

    Vibration      NUE*CM                                      NUE*CM
       Nr           [160]              [180]                   [160]               [180]

                                                         >> AVDZ:
       1                                               A1   433.26              430.06
       2                                               B2   772.85              747.50
       3                                               A1   875.78              847.82
     ZPE                                                   1054.28             1025.27

                                                         >> AVTZ:
       1                                               A1   458.35              454.79
       2                                               B2   840.58              812.93
       3                                               A1   931.98              902.50
     ZPE                                                   1128.69             1097.59
```

Ref.: $^{16}OF_2$ gas phase IR spectrum: ν_1 928/cm (A_1, sym str, strong), ν_2 461/cm (A_1, bend, very strong), ν_3 831/cm (B_2, antisym str, strong) [13]

Quantum-Chemical Results for the Molecule AuOF

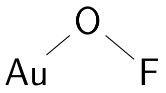

Results for Au-O-F X 1-A'
=========================

Closed-shell system, Au ECP60MDF, 36 electrons
Electron configuration: a' 1-13, a" 1-5
Type and number of normal modes: A' 1-3

>> RHF:
Optimized structural parameters, associated total energy, dipole moment:

BASIS	AU-O/ANG	O-F/ANG	AU-O-F/DEG	EHF/AU	MU/D
VDZ(-PP)	2.04273	1.40456	104.717	-308.91877350	6.3921
AVDZ(-PP)	2.05055	1.40396	104.864	-308.93556304	6.4258
VTZ(-PP)	2.03155	1.39208	106.238	-308.97731164	6.2229
AVTZ(-PP)	2.02915	1.39316	106.288	-308.98150569	6.1961

Normal mode analysis (harmonic frequencies and relative IR intensities):

Vibration Nr		WE*CM [160]		[180]			WE*CM [160]		[180]	
		>> VDZ(-PP):					>> AVDZ(-PP):			
1	A'	210.79	(13.63)	208.83	(12.61)	A'	206.69	(12.40)	204.79	(11.59)
2	A'	501.68	(89.90)	475.76	(80.37)	A'	494.14	(78.79)	468.59	(70.77)
3	A'	1035.19	(100.00)	1003.26	(100.00)	A'	1034.37	(100.00)	1002.42	(100.00)
ZPE		873.83		843.93			867.61		837.90	
		>> VTZ(-PP):					>> AVTZ(-PP):			
1	A'	215.85	(13.39)	213.82	(12.50)	A'	213.89	(14.09)	211.97	(13.20)
2	A'	505.42	(84.82)	479.32	(76.09)	A'	502.64	(81.56)	476.67	(73.19)
3	A'	1085.17	(100.00)	1051.53	(100.00)	A'	1078.92	(100.00)	1045.46	(100.00)
ZPE		903.22		872.33			897.77		867.05	

>> B3LYP [GAUSSIAN]:
Optimized structural parameters, associated total energy, dipole moment:

BASIS	AU-O/ANG	O-F/ANG	AU-O-F/DEG	EDFT/AU	MU/D
AVTZ(-PP)	1.98008	1.43873	108.098	-310.75736412	3.8685

Normal mode analysis (harmonic frequencies, absolute IR intensities in km/mol):

Vibration Nr		WE*CM [160]		[180]			WE*CM [160]		[180]	
							>> AVTZ(-PP):			
1						A'	228.55	(4.91)	226.28	(4.59)
2						A'	521.09	(10.55)	493.77	(9.27)
3						A'	900.92	(89.22)	873.50	(86.08)
ZPE							825.28		796.77	

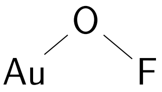

Results for Au-O-F X 1-A' (continued)
========================
Closed-shell system, Au ECP60MDF, 36 electrons
Electron configuration: a' 1-13, a" 1-5
Type and number of normal modes: A' 1-3

>> CCSD(T):
Optimized structural parameters, associated total energy:

BASIS	AU-O/ANG	O-F/ANG	AU-O-F/DEG	ECC/AU	T1(+)
VDZ(-PP)	1.99262	1.47886	104.604	-309.61797021	0.0433
AVDZ(-PP)	2.00584	1.48709	104.320	-309.69450418	0.0445
VTZ(-PP)	1.97609	1.45321	105.730	-309.87831478	0.0391
AVTZ(-PP)	1.97566	1.46051	105.554	-309.90881884	0.0384

(+) T1 diagnostic from CCSD(T)

Normal mode analysis (harmonic frequencies):

Vibration Nr		WE*CM [160]	[180]		WE*CM [160]	[180]
		>> VDZ(-PP):			>> AVDZ(-PP):	
1	A'	217.32	215.20	A'	210.80	208.80
2	A'	509.55	482.93	A'	500.40	474.22
3	A'	762.77	739.53	A'	769.37	745.87
ZPE		744.82	718.83		740.28	714.44
		>> VTZ(-PP):			>> AVTZ(-PP):	
1	A'	224.03	221.84	A'	220.01	217.88
2	A'	523.28	495.97	A'	523.68	496.38
3	A'	846.75	820.82	A'	832.22	806.66
ZPE		797.03	769.13		787.95	760.46

Results from VSCF calculation on CCSD(T) PES:

Vibration Nr		NUE*CM [160]	[180]		NUE*CM [160]	[180]
					>> AVTZ(-PP):	
1				A'	217.90	215.90
2				A'	516.51	489.85
3				A'	810.40	786.17
ZPE					783.52	756.32

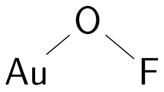

```
Results for Au-O-F X 1-A' (continued)
=========================
Closed-shell system, Au ECP60MDF, 36 electrons
Electron configuration: a' 1-13, a" 1-5
Type and number of normal modes: A' 1-3

>> CCSD(T) [CFOUR]:
Optimized structural parameters, associated total energy:

BASIS        AU-O/ANG   O-F/ANG  AU-O-F/DEG        ECC/AU
AVTZ(-PP)     1.97564   1.46048     105.550   -309.90881885

Normal mode analysis (harmonic frequencies, absolute IR intensities in km/mol):

    Vibration       WE*CM                                        WE*CM
       Nr           [16O]              [18O]                     [16O]              [18O]

                                                              >> AVTZ(-PP):
       1                                                   A'   220.30 (   5.40)    218.16 (   5.06)
       2                                                   A'   523.80 (  11.82)    496.48 (  10.40)
       3                                                   A'   832.47 (  38.76)    806.91 (  38.05)
     ZPE                                                        788.28              760.78
```

Quantum-Chemical Results for the Molecule OAuF

O — Au — F

Results for O-Au-F X 3-Sigma- [3-A2]
====================================
Open-shell system, Au ECP60MDF, 36 electrons
Electron configuration:
- closed-shell part: sigma 1-9, pi 1-3, delta 1 [a1 1-10, a2 1, b1 1-3, b2 1-3]
- open-shell part: (4pi)2 [4(b1 & b2)2]

Type and number of normal modes: Sigma (A) 1-2, Pi (E1) 1 [A1 1-2, B1 1, B2 1]

>> RHF (minimal sa-MCSCF for (4pi)2 in C2v or C1, with the only non-zero weight on 3-Sigma-):
Optimized structural parameters, associated total energy, dipole moment:

BASIS	AU-O/ANG	AU-F/ANG	EHF/AU	MU/D
VDZ(-PP)	2.10757	1.93515	-308.99786317	5.9640
AVDZ(-PP)	2.07250	1.94169	-309.01659169	6.0256
VTZ(-PP)	2.04534	1.92271	-309.05383839	5.8076
AVTZ(-PP)	2.03333	1.92191	-309.05927604	5.7757

Normal mode analysis (harmonic frequencies and relative IR intensities):

Vibration Nr		WE*CM [160]	[180]		WE*CM [160]	[180]
		>> VDZ(-PP):			>> AVDZ(-PP):	
1	B1	147.95 (7.04)	144.41 (7.78)	B1	159.26 (6.44)	155.37 (7.05)
2	B2	147.95 (7.04)	144.41 (7.78)	B2	159.26 (6.44)	155.37 (7.05)
3	A1	273.72 (17.81)	259.29 (15.61)	A1	307.74 (26.95)	291.62 (23.33)
4	A1	604.51 (100.00)	604.16 (100.00)	A1	592.58 (100.00)	592.03 (100.00)
ZPE		587.06	576.13		609.42	597.19
		>> VTZ(-PP):			>> AVTZ(-PP):	
1	B1	165.66 (5.77)	161.60 (6.35)	B1	169.20 (5.78)	165.02 (6.33)
2	B2	165.66 (5.77)	161.60 (6.35)	B2	169.20 (5.78)	165.02 (6.33)
3	A1	320.76 (30.20)	303.97 (26.11)	A1	331.46 (34.93)	314.13 (30.06)
4	A1	607.57 (100.00)	607.00 (100.00)	A1	603.13 (100.00)	602.51 (100.00)
ZPE		629.83	617.08		636.49	623.34

Results from VSCF calculation on RHF PES:

Vibration Nr		NUE*CM [160]	[180]		NUE*CM [160]	[180]
		>> VDZ(-PP):			>> AVDZ(-PP):	
1	B1	143.07	139.86	B1	154.42	150.86
2	B2	143.07	139.86	B2	154.42	150.86
3	A1	259.60	246.46	A1	292.99	278.26
4	A1	595.62	595.37	A2	583.35	582.92
ZPE		581.10	570.56		602.92	591.11
		>> VTZ(-PP):			>> AVTZ(-PP):	
1	B1	160.60	156.87	B1	164.06	160.22
2	B2	160.60	156.87	B2	164.06	160.22
3	A1	304.48	289.16	A1	314.36	298.58
4	A1	598.11	597.67	A1	593.25	592.76
ZPE		623.22	610.90		629.62	616.92

```
Results for O-Au-F X 3-Sigma- [3-A2] (continued)
====================================
Open-shell system, Au ECP60MDF, 36 electrons
Electron configuration:
- closed-shell part: sigma 1-9, pi 1-3, delta 1 [a1 1-10, a2 1, b1 1-3, b2 1-3]
-   open-shell part: (4pi)2 [4(b1 & b2)2]
Type and number of normal modes: Sigma (A) 1-2, Pi (E1) 1 [A1 1-2, B1 1, B2 1]

>> B3LYP [GAUSSIAN]:
Optimized structural parameters, associated total energy, dipole moment:

BASIS       AU-O/ANG  AU-F/ANG        EDFT/AU      MU/D
AVTZ(-PP)    1.81154   1.89011   -310.82776696   1.4688

Normal mode analysis (harmonic frequencies, absolute IR intensities in km/mol):

    Vibration       WE*CM                                     WE*CM
       Nr           [160]              [180]                  [160]              [180]

                                                           >> AVTZ(-PP):
       1                                               B1    190.61 (  4.93)    185.49 (  4.87)
       2                                               B2    190.61 (  4.93)    185.49 (  4.87)
       3                                               A1    628.50 ( 81.73)    623.65 ( 79.71)
       4                                               A1    757.70 (  2.66)    722.93 (  7.48)
     ZPE                                                     883.71             858.78

>> CISD:
Optimized structural parameters, associated total energy, dipole moment:

BASIS       AU-O/ANG  AU-F/ANG         ECI/AU      MU/D
VDZ(-PP)     1.85682   1.89741   -309.56339136   4.4160
AVDZ(-PP)    1.85044   1.90781   -309.62198744   4.5459
VTZ(-PP)     1.82692   1.88407   -309.77357389   4.3453
AVTZ(-PP)    1.82623   1.88548   -309.79627855   4.3523

Normal mode analysis (harmonic frequencies and relative IR intensities):

    Vibration       WE*CM                                     WE*CM
       Nr           [160]              [180]                  [160]              [180]

                >> VDZ(-PP):                               >> AVDZ(-PP):
       1      B1    187.50 (  0.00)    182.53 (  0.00)    B1    195.31 (  0.00)    190.10 (  0.00)
       2      B2    187.50 (  0.00)    182.53 (  0.00)    B2    195.31 (  0.00)    190.10 (  0.00)
       3      A1    516.29 (100.00)    490.72 (100.00)    A1    521.57 (100.00)    496.69 (100.00)
       4      A1    670.37 ( 33.52)    667.74 ( 47.46)    A1    659.49 ( 23.81)    655.64 ( 37.40)
     ZPE            780.83             761.76                   785.84             766.26

                >> VTZ(-PP):                               >> AVTZ(-PP):
       1      B1    199.51 (  0.00)    194.18 (  0.00)    B1    200.78 (  0.00)    195.41 (  0.00)
       2      B2    199.51 (  0.00)    194.18 (  0.00)    B2    200.78 (  0.00)    195.41 (  0.00)
       3      A1    551.85 (100.00)    526.08 (100.00)    A1    557.37 (100.00)    531.98 (100.00)
       4      A1    681.02 ( 19.61)    676.33 ( 33.50)    A1    677.72 ( 15.22)    672.25 ( 28.67)
     ZPE            815.94             795.38                   818.32             797.53
```

Results for O-Au-F X 3-Sigma- [3-A2] (continued)
====================================
Open-shell system, Au ECP60MDF, 36 electrons
Electron configuration:
- closed-shell part: sigma 1-9, pi 1-3, delta 1 [a1 1-10, a2 1, b1 1-3, b2 1-3]
- open-shell part: (4pi)2 [4(b1 & b2)2]
Type and number of normal modes: Sigma (A) 1-2, Pi (E1) 1 [A1 1-2, B1 1, B2 1]

>> RCCSD(T):
Optimized structural parameters, associated total energy:

BASIS	AU-O/ANG	AU-F/ANG	ECC/AU	T1(+)
VDZ(-PP)	1.82569	1.89367	-309.67755128	0.0544
AVDZ(-PP)	1.82580	1.90512	-309.75636888	0.0550
VTZ(-PP)	1.80341	1.87965	-309.93572409	0.0520
AVTZ(-PP)	1.80428	1.88266	-309.96778827	0.0522
VQZ(-PP)	1.79859	1.87756	-310.02696446	0.0508
AVQZ(-PP)	1.79879	1.87865	-310.04036125	0.0509

(+) T1 diagnostic from RCCSD(T)

Normal mode analysis (harmonic frequencies):

Vibration Nr		WE*CM [160]	[180]		WE*CM [160]	[180]
		>> VDZ(-PP):			>> AVDZ(-PP):	
1	B1	181.75	176.83	B1	189.54	184.47
2	B2	181.75	176.83	B2	189.54	184.47
3	A1	637.22	627.68	A1	623.17	615.40
4	A1	739.53	710.79	A1	733.84	703.54
ZPE		870.13	846.07		868.05	843.94
		>> VTZ(-PP):			>> AVTZ(-PP):	
1	B1	190.07	184.96	B1	191.38	186.23
2	B2	190.07	184.96	B2	191.38	186.23
3	A1	645.30	638.99	A1	639.17	633.84
4	A1	770.56	736.73	A1	771.36	736.41
ZPE		898.01	872.82		896.65	871.36
		>> VQZ(-PP):			>> AVQZ(-PP):	
1	B1	200.82	195.41	B1	197.31	192.09
2	B2	200.82	195.41	B2	197.31	192.09
3	A1	648.77	643.22	A1	645.16	639.87
4	A1	778.25	743.16	A1	777.75	742.42
ZPE		914.33	888.60		908.77	883.23

O—Au—F

Results for O-Au-F X 3-Sigma- [3-A2] (continued)
===================================
Open-shell system, Au ECP60MDF, 36 electrons
Electron configuration:
- closed-shell part: sigma 1-9, pi 1-3, delta 1 [a1 1-10, a2 1, b1 1-3, b2 1-3]
- open-shell part: (4pi)2 [4(b1 & b2)2]
Type and number of normal modes: Sigma (A) 1-2, Pi (E1) 1 [A1 1-2, B1 1, B2 1]

>> RCCSD(T) (continued):
Results from VSCF calculation on RCCSD(T) PES:

Vibration Nr		NUE*CM [16O]	[18O]		NUE*CM [16O]	[18O]
		>> VDZ(-PP):			>> AVDZ(-PP):	
1	B1	180.26	175.43	B1	186.73	181.35
2	B2	180.26	175.44	B2	186.73	181.36
3	A1	630.65	621.37	A1	615.59	608.37
4	A1	728.09	701.53	A1	722.88	694.42
ZPE		866.84	843.23		863.08	839.09
		>> VTZ(-PP):			>> AVTZ(-PP):	
1	B1	190.53	185.40	B1	191.05	185.92
2	B2	190.53	185.40	B2	191.05	185.92
3	A1	640.34	634.54	A1	633.65	628.76
4	A1	760.46	728.20	A1	760.97	727.58
ZPE		898.31	873.36		895.95	870.91
		>> VQZ(-PP):			>> AVQZ(-PP):	
1	B1	191.29	186.14			
2	B2	191.30	186.14			
3	A1	641.34	636.16			
4	A1	766.74	733.41			
ZPE		902.79	877.61			

>> RCCSD(T) [CFOUR]:
Optimized structural parameters, associated total energy:

BASIS	AU-O/ANG	AU-F/ANG	ECC/AU
AVTZ(-PP)	1.81059	1.88184	-309.97133230

Normal mode analysis (harmonic frequencies, absolute IR intensities in km/mol):

Vibration Nr		WE*CM [16O]	[18O]		WE*CM [16O]	[18O]
					>> AVTZ(-PP):	
1				B1	192.22 (5.10)	187.03 (5.08)
2				B2	192.22 (5.10)	187.03 (5.08)
3				A1	642.38 (88.05)	637.12 (79.75)
4				A1	767.87 (8.92)	732.98 (17.21)
ZPE					897.34	872.08

Quantum-Chemical Results for the Molecule AgOF

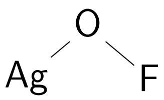

Results for Ag-O-F X 1-A'
=========================

Closed-shell system, Ag ECP28MDF, 36 electrons
Electron configuration: a' 1-13, a" 1-5
Type and number of normal modes: A' 1-3

Optimized structural parameters, associated total energy, and dipole moment:

BASIS	METHOD	AG-O/ANG	O-F/ANG	AG-O-F/DEG	E/AU	MU/D
AVTZ(-PP)	B3LYP [GAUSSIAN]	2.05941	1.45265	104.985	-321.98718833	5.6564
AVTZ(-PP)	CCSD(T) [CFOUR]	2.06062	1.48245	98.932	-321.21061482	--

Normal mode analysis (harmonic frequencies, absolute IR intensities in km/mol):

Vibration Nr		WE*CM [16O/107AG]	[18O/107AG]	[16O/109AG]	[18O/109AG]
>> B3LYP [GAUSSIAN]:					
		>> AVTZ(-PP):			
1	A'	172.48 (5.91)	170.83 (5.49)	172.23 (5.88)	170.59 (5.47)
2	A'	457.84 (19.54)	435.31 (17.54)	457.35 (19.47)	434.79 (17.47)
3	A'	905.07 (69.47)	877.69 (67.80)	905.06 (69.48)	877.69 (67.81)
ZPE		767.69	741.92	767.32	741.54
>> CCSD(T) [CFOUR]:					
		>> AVTZ(-PP):			
1	A'	149.37 (6.52)	148.00 (6.08)	149.16 (6.49)	147.80 (6.05)
2	A'	471.60 (26.64)	448.50 (24.13)	471.05 (26.55)	447.92 (24.04)
3	A'	831.60 (17.50)	806.65 (18.06)	831.60 (17.50)	806.65 (18.06)
ZPE		726.28	701.58	725.90	701.19

Quantum-Chemical Results for the Molecule OAgF

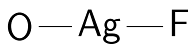

Results for O-Ag-F X 3-Sigma- [3-A2]
=====================================

Open-shell system, Ag ECP28MDF, 36 electrons
Electron configuration:
- closed-shell part: sigma 1-9, pi 1-3, delta 1 [a1 1-10, a2 1, b1 1-3, b2 1-3]
- open-shell part: (4pi)2 [4(b1 & b2)2]

Type and number of normal modes: Sigma (A) 1-2, Pi (E1) 1 [A1 1-2, B1 1, B2 1]

Optimized structural parameters, associated total energy, and dipole moment:

BASIS	METHOD	AG-O/ANG	AG-F/ANG	E/AU	MU/D
AVTZ(-PP)	B3LYP [GAUSSIAN]	1.88791	1.91327	-322.01452857	2.5957
AVTZ(-PP)	CCSD(T) [CFOUR]	1.89416	1.91093	-321.22180061	--

Normal mode analysis (harmonic frequencies, absolute IR intensities in km/mol):

Vibration Nr		WE*CM [16O/107AG]	[18O/107AG]	[16O/109AG]	[18O/109AG]
>> B3LYP [GAUSSIAN]:					
		>> AVTZ(-PP):			
1	B1	131.85 (11.91)	128.81 (11.87)	131.55 (11.85)	128.51 (11.82)
2	B2	131.85 (11.91)	128.81 (11.87)	131.55 (11.85)	128.51 (11.82)
3	A1	506.78 (64.64)	490.02 (55.65)	506.78 (64.65)	490.01 (55.59)
4	A1	649.80 (14.78)	637.96 (22.36)	648.33 (14.70)	636.47 (22.34)
ZPE		710.13	692.80	709.10	691.75
>> CCSD(T) [CFOUR]:					
		>> AVTZ(-PP):			
1	B1	127.12 (12.73)	124.16 (12.86)	126.84 (12.66)	123.87 (12.80)
2	B2	127.12 (12.73)	124.16 (12.86)	126.84 (12.66)	123.87 (12.80)
3	A1	484.96 (155.13)	466.96 (138.36)	484.95 (155.08)	466.92 (138.22)
4	A1	647.97 (12.89)	638.83 (22.77)	646.51 (12.91)	637.38 (22.88)
ZPE		693.59	677.06	692.57	676.02

Quantum-Chemical Results for the Molecule CuOF

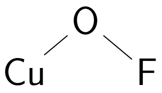

Results for Cu-O-F X 1-A'
=========================

Closed-shell system, Cu ECP10MDF, 36 electrons
Electron configuration: a' 1-13, a" 1-5
Type and number of normal modes: A' 1-3

Optimized structural parameters, associated total energy, and dipole moment:

BASIS	METHOD	CU-O/ANG	O-F/ANG	CU-O-F/DEG	E/AU	MU/D
AVTZ(-PP)	B3LYP [GAUSSIAN]	1.79971	1.45848	105.107	-372.36358539	5.0695
AVTZ(-PP)	CCSD(T) [CFOUR]	1.80040	1.48385	100.535	-371.44375993	--

Normal mode analysis (harmonic frequencies, absolute IR intensities in km/mol):

Vibration Nr		WE*CM [16O/63CU]	[18O/63CU]	[16O/65CU]	[18O/65CU]
>> B3LYP [GAUSSIAN]:					
	>> AVTZ(-PP):				
1	A'	203.46 (9.32)	201.11 (8.74)	202.78 (9.22)	200.44 (8.64)
2	A'	569.49 (22.45)	544.18 (20.24)	567.78 (22.25)	542.38 (20.04)
3	A'	880.83 (56.73)	854.15 (56.06)	880.82 (56.76)	854.15 (56.08)
ZPE		826.89	799.72	825.69	798.49
>> CCSD(T) [CFOUR]:					
	>> AVTZ(-PP):				
1	A'	177.33 (11.22)	175.32 (10.90)	176.75 (11.15)	174.75 (10.84)
2	A'	572.39 (29.75)	547.17 (27.28)	570.61 (29.48)	545.30 (27.02)
3	A'	820.42 (10.00)	795.66 (10.48)	820.40 (10.03)	795.64 (10.51)
ZPE		785.07	759.07	783.88	757.84

Quantum-Chemical Results for the Molecule OCuF

O — Cu — F

```
Results for O-Cu-F X 3-Sigma- [3-A2]
====================================
Open-shell system, Cu ECP10MDF, 36 electrons
Electron configuration:
- closed-shell part: sigma 1-9, pi 1-3, delta 1 [a1 1-10, a2 1, b1 1-3, b2 1-3]
-   open-shell part: (4pi)2 [4(b1 & b2)2]
Type and number of normal modes: Sigma (A) 1-2, Pi (E1) 1 [A1 1-2, B1 1, B2 1]

Optimized structural parameters, associated total energy, and dipole moment:

BASIS       METHOD            CU-O/ANG   CU-F/ANG         E/AU         MU/D

AVTZ(-PP)   B3LYP [GAUSSIAN]   1.66699    1.71643    -372.44412336   1.3938
AVTZ(-PP)   CCSD(T) [CFOUR]    1.69512    1.70633    -371.52720639     --

Normal mode analysis (harmonic frequencies, absolute IR intensities in km/mol):

    Vibration       WE*CM
       Nr           [16O/63CU]          [18O/63CU]          [16O/65CU]          [18O/65CU]

>> B3LYP [GAUSSIAN]:
                 >> AVTZ(-PP):
       1      B1    160.20 ( 21.13)    156.92 ( 20.67)    159.32 ( 20.89)    156.03 ( 20.43)
       2      B2    160.20 ( 21.13)    156.92 ( 20.67)    159.32 ( 20.89)    156.03 ( 20.43)
       3      A1    633.24 ( 59.24)    619.54 ( 52.68)    633.02 ( 59.41)    619.48 ( 52.81)
       4      A1    837.79 ( 14.54)    815.44 ( 20.75)    833.45 ( 14.03)    810.87 ( 20.28)
     ZPE            895.71             874.42             892.56             871.20

>> CCSD(T) [CFOUR]:
                 >> AVTZ(-PP):
       1      B1    162.90 ( 28.13)    159.68 ( 27.26)    162.01 ( 27.82)    158.77 ( 26.95)
       2      B2    162.90 ( 28.13)    159.68 ( 27.26)    162.01 ( 27.82)    158.77 ( 26.95)
       3      A1    659.10 (  0.53)    644.63 (  5.65)    658.87 (  0.40)    644.57 (  5.42)
       4      A1    837.47 (455.18)    815.41 (423.28)    833.14 (450.69)    810.82 (418.89)
     ZPE            911.19             889.70             908.02             886.46
```

Quantum-Chemical Results for the Molecule AuF(OF)

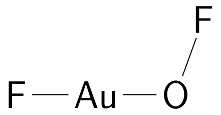

Results for F-Au-O-F X 2-A"
===========================

Open-shell system, Au ECP60MDF, 45 electrons
Electron configuration:
- closed-shell part: a’ 1-17, a" 1-5
- open-shell part: (6a")1

Type and number of normal modes: A’ 1-5, A" 1

>> RHF:
Optimized structural parameters, associated total energy, dipole moment:

BASIS	AU-O/ANG	O-F/ANG	AU-F/ANG	AU-O-F/DEG	F-AU-O/DEG	TAU/DEG
VDZ(-PP)	1.91543	1.37976	1.89486	110.346	182.005	0.000
AVDZ(-PP)	1.91666	1.37897	1.89816	109.958	182.002	0.000
VTZ(-PP)	1.90224	1.36952	1.88360	111.358	182.172	0.000
AVTZ(-PP)	1.90123	1.37060	1.88295	111.195	182.124	0.000

BASIS	EHF/AU	MU/D
VDZ(-PP)	-408.31386626	0.4765
AVDZ(-PP)	-408.34414061	0.5442
VTZ(-PP)	-408.41119710	0.4738
AVTZ(-PP)	-408.41836706	0.4980

Normal mode analysis (harmonic frequencies and relative IR intensities):

Vibration Nr		WE*CM [160]	[180]		WE*CM [160]	[180]
		>> VDZ(-PP):			>> AVDZ(-PP):	
1	A"	71.55 (9.91)	69.96 (11.03)	A"	91.14 (8.97)	89.10 (9.88)
2	A’	145.18 (4.67)	145.16 (5.38)	A’	146.32 (4.28)	146.29 (4.86)
3	A’	296.43 (2.95)	292.53 (3.20)	A’	295.95 (3.39)	292.12 (3.62)
4	A’	593.76 (17.16)	567.41 (27.80)	A’	589.64 (11.37)	563.50 (20.52)
5	A’	671.23 (100.00)	667.12 (100.00)	A’	663.51 (100.00)	659.21 (100.00)
6	A’	1118.46 (10.49)	1081.72 (12.78)	A’	1126.34 (13.91)	1089.48 (16.33)
ZPE		1448.31	1411.95		1456.45	1419.85
		>> VTZ(-PP):			>> AVTZ(-PP):	
1	A"	90.88 (8.17)	89.04 (9.06)	A"	93.84 (7.87)	90.97 (8.75)
2	A’	149.24 (3.87)	149.23 (4.43)	A’	149.06 (3.73)	149.04 (4.27)
3	A’	304.47 (2.93)	300.47 (3.15)	A’	303.62 (3.14)	299.65 (3.40)
4	A’	600.88 (10.89)	574.66 (20.82)	A’	602.86 (8.10)	577.00 (18.10)
5	A’	673.23 (100.00)	668.52 (100.00)	A’	670.60 (100.00)	665.34 (100.00)
6	A’	1170.64 (12.68)	1132.15 (15.06)	A’	1165.88 (13.03)	1227.54 (15.48)
ZPE		1494.67	1457.04		1492.93	1454.77

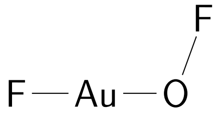

Results for F-Au-O-F X 2-A" (continued)
===========================

Open-shell system, Au ECP60MDF, 45 electrons
Electron configuration:
- closed-shell part: a’ 1-17, a" 1-5
-   open-shell part: (6a")1

Type and number of normal modes: A’ 1-5, A" 1

>> B3LYP [GAUSSIAN]:
Optimized structural parameters, associated total energy, dipole moment:

BASIS	AU-O/ANG	O-F/ANG	AU-F/ANG	AU-O-F/DEG	F-AU-O/DEG	TAU/DEG
AVTZ(-PP)	1.89913	1.41527	1.89398	113.637	176.318	180.000

BASIS	EDFT/AU	MU/D
AVTZ(-PP)	-410.64241705	1.6120

Normal mode analysis (harmonic frequencies, absolute IR intensities in km/mol):

Vibration Nr	WE*CM [160]	[180]		WE*CM [160]	[180]
				>> AVTZ(-PP):	
1			A"	142.96 (4.55)	139.82 (4.58)
2			A’	140.18 (3.18)	140.17 (3.18)
3			A’	300.91 (0.64)	296.91 (0.61)
4			A’	586.55 (34.13)	563.70 (18.67)
5			A’	654.18 (58.24)	644.96 (73.86)
6			A’	894.17 (172.72)	866.44 (161.49)
ZPE				1359.47	1326.00

>> CCSD(T) [CFOUR]:
Optimized structural parameters, associated total energy:

BASIS	AU-O/ANG	O-F/ANG	AU-F/ANG	AU-O-F/DEG	F-AU-O/DEG	TAU/DEG
AVTZ(-PP)	1.87738	1.42972	1.88401	112.196	176.701	180.000

BASIS	ECC/AU
AVTZ(-PP)	-409.64205330

Normal mode analysis (harmonic frequencies, absolute IR intensities in km/mol):

Vibration Nr	WE*CM [160]	[180]		WE*CM [160]	[180]
				>> AVTZ(-PP):	
1			A"	141.58 (4.71)	138.32 (4.49)
2			A’	148.03 (3.69)	148.02 (3.69)
3			A’	302.64 (0.89)	298.72 (0.85)
4			A’	614.14 (48.44)	593.14 (24.48)
5			A’	679.70 (82.96)	667.63 (107.23)
6			A’	835.44 (127.85)	808.25 (118.43)
ZPE				1360.77	1327.04

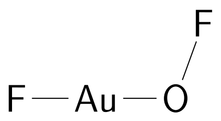

Results for F-Au-O-F X 2-A" (continued)
===========================
Open-shell system, Au ECP60MDF, 45 electrons
Electron configuration:
- closed-shell part: a' 1-17, a" 1-5
- open-shell part: (6a")1
Type and number of normal modes: A' 1-5, A" 1

>> RCCSD(T):
Optimized structural parameters, associated total energy:

BASIS	AU-O/ANG	O-F/ANG	AU-F/ANG	AU-O-F/DEG	F-AU-O/DEG	TAU/DEG
VDZ(-PP)	1.88559	1.45201	1.89370	111.460	183.324	0.000
AVDZ(-PP)	1.88550	1.45817	1.90387	111.169	183.339	0.000
VTZ(-PP)	1.86516	1.42641	1.87945	112.585	183.320	0.000
AVTZ(-PP)	1.86434	1.43272	1.88172	112.304	183.268	0.000

BASIS	ECC/AU	T1(+)
VDZ(-PP)	-409.23438710	0.0512
AVDZ(-PP)	-409.34161362	0.0518
VTZ(-PP)	-409.59617425	0.0463
AVTZ(-PP)	-409.63832250	0.0464

(+) T1 diagnostic from CCSD(T)

Normal mode analysis (harmonic frequencies):

Vibration Nr		WE*CM [160]	[180]		WE*CM [160]	[180]
		>> VDZ(-PP):			>> AVDZ(-PP):	
1	A"	124.30	121.46	A"	136.16	133.00
2	A'	140.04	139.92	A'	139.24	139.19
3	A'	299.23	295.61	A'	296.59	292.83
4	A'	613.62	591.33	A'	604.26	584.41
5	A'	674.95	665.41	A'	665.47	653.29
6	A'	764.36	738.34	A'	772.33	746.37
ZPE		1308.25	1276.03		1307.02	1274.54
		>> VTZ(-PP):			>> AVTZ(-PP):	
1	A"	136.53	133.22	A"	141.54	138.22
2	A'	143.42	143.28	A'	145.67	145.59
3	A'	308.93	304.69	A'	312.80	308.49
4	A'	628.94	610.00	A'	628.87	612.91
5	A'	689.56	675.34	A'	688.54	671.64
6	A'	857.19	828.14	A'	844.96	816.08
ZPE		1382.29	1347.33		1381.19	1346.46

Quantum-Chemical Results for the Molecule $Au(O)F_2$

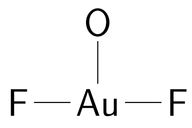

Results for O-Au(-F)2 X 2-B2
============================
Open-shell system, Au ECP60MDF, 45 electrons
Electron configuration:
- closed-shell part: a1 1-10, a1 1-2, b1 1-4, b2 1-6
- open-shell part: (7b2)1

Type and number of normal modes: A1 1-3, B1 1, B2 1-2

>> RHF:
Optimized structural parameters, associated total energy, dipole moment:

BASIS	AU-O/ANG	AU-F/ANG	O-AU-F/DEG	EHF/AU	MU/D
VDZ(-PP)	1.88222	1.88641	92.845	-408.35206616	2.1111
AVDZ(-PP)	1.88250	1.88701	92.952	-408.38319745	2.0580
VTZ(-PP)	1.87059	1.87668	92.869	-408.44789492	2.0828
AVTZ(-PP)	1.86855	1.87569	92.879	-408.45642924	2.0577

Normal mode analysis (harmonic frequencies and relative IR intensities):

Vibration Nr		WE*CM [160]	[180]		WE*CM [160]	[180]
		>> VDZ(-PP):			>> AVDZ(-PP):	
1	B2	195.35 (0.28)	187.93 (0.23)	B2	194.81 (0.25)	187.41 (0.20)
2	B1	228.64 (11.34)	228.57 (11.27)	B1	231.01 (9.72)	230.94 (9.66)
3	A1	231.10 (9.46)	230.91 (9.43)	A1	231.69 (8.09)	231.49 (8.06)
4	A1	661.00 (5.89)	639.15 (4.66)	A1	658.01 (6.56)	634.82 (5.16)
5	A1	694.49 (0.01)	680.34 (1.14)	A1	692.19 (0.00)	679.62 (1.26)
6	B2	700.38 (100.00)	700.37 (100.00)	B2	693.58 (100.00)	693.57 (100.00)
ZPE		1355.48	1333.63		1350.64	1328.92
		>> VTZ(-PP):			>> AVTZ(-PP):	
1	B2	196.61 (0.34)	189.14 (0.28)	B2	197.61 (0.33)	190.10 (0.27)
2	B1	234.21 (9.65)	234.15 (9.59)	B1	233.99 (9.37)	233.93 (9.31)
3	A1	235.23 (8.01)	235.03 (7.98)	A1	235.13 (7.82)	234.93 (7.79)
4	A1	666.59 (6.68)	646.85 (5.96)	A1	666.67 (6.57)	648.83 (6.11)
5	B2	699.88 (100.00)	699.87 (100.00)	B2	696.37 (100.00)	696.36 (100.00)
6	A1	705.27 (0.16)	688.46 (0.70)	A1	708.75 (0.24)	689.82 (0.52)
ZPE		1368.90	1346.75		1369.26	1346.98

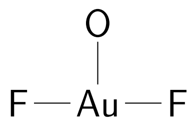

Results for O-Au(-F)2 X 2-B2 (continued)
============================

Open-shell system, Au ECP60MDF, 45 electrons
Electron configuration:
- closed-shell part: a1 1-10, a1 1-2, b1 1-4, b2 1-6
- open-shell part: (7b2)1

Type and number of normal modes: A1 1-3, B1 1, B2 1-2

>> B3LYP [GAUSSIAN]:
Optimized structural parameters, associated total energy, dipole moment:

BASIS	AU-O/ANG	AU-F/ANG	O-AU-F/DEG	EDFT/AU	MU/D
AVTZ(-PP)	1.85725	1.91288	96.275	-410.64034577	1.1010

Normal mode analysis (harmonic frequencies, absolute IR intensities in km/mol):

Vibration Nr	WE*CM [160]	[180]		WE*CM [160]	[180]
				>> AVTZ(-PP):	
1			B2	142.95 (2.02)	137.48 (1.80)
2			A1	183.86 (6.22)	183.81 (6.22)
3			B1	205.71 (11.04)	205.45 (10.90)
4			A1	632.52 (0.60)	599.88 (0.47)
5			A1	597.77 (5.17)	596.71 (5.18)
6			B2	637.45 (141.41)	637.44 (141.47)
ZPE				1200.13	1180.38

>> RCCSD(T) [CFOUR]:
Optimized structural parameters, associated total energy, dipole moment:

BASIS	AU-O/ANG	AU-F/ANG	O-AU-F/DEG	ECC/AU	MU/D
AVTZ(-PP)	1.86130	1.90043	94.846	-409.64670803	--

Normal mode analysis (harmonic frequencies, absolute IR intensities in km/mol):

Vibration Nr	WE*CM [160]	[180]		WE*CM [160]	[180]
				>> AVTZ(-PP):	
1			B2	147.98 (0.69)	142.32 (0.60)
2			A1	191.85 (8.42)	191.79 (8.42)
3			B1	210.87 (11.91)	210.70 (11.82)
4			A1	617.69 (0.00)	585.64 (1.77)
5			A1	620.32 (6.39)	619.40 (4.32)
6			B2	659.17 (146.58)	659.17 (146.61)
ZPE				1223.94	1204.51

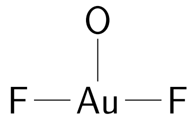

Results for O-Au(-F)2 X 2-B2 (continued)
============================

Open-shell system, Au ECP60MDF, 45 electrons
Electron configuration:
- closed-shell part: a1 1-10, a1 1-2, b1 1-4, b2 1-6
- open-shell part: (7b2)1

Type and number of normal modes: A1 1-3, B1 1, B2 1-2

>> RCCSD(T):
Optimized structural parameters, associated total energy:

BASIS	AU-O/ANG	AU-F/ANG	O-AU-F/DEG	ECC/AU	T1(+)
VDZ(-PP)	1.88889	1.91357	94.621	-409.24121842	0.0309
AVDZ(-PP)	1.89283	1.92022	94.526	-409.35003382	0.0323
VTZ(-PP)	1.86331	1.89929	94.720	-409.60135752	0.0303
AVTZ(-PP)	1.86372	1.90074	94.614	-409.64573391	0.0308

(+) T1 diagnostic from RCCSD(T)

Normal mode analysis (harmonic frequencies):

Vibration Nr		WE*CM [16O]	[18O]		WE*CM [16O]	[18O]
		>> VDZ(-PP):			>> AVDZ(-PP):	
1	B2	149.94	144.23	B2	144.18	138.21
2	A1	191.29	191.22	A1	190.28	189.87
3	B1	203.78	203.63	B1	205.55	205.16
4	A1	580.24	549.37	A1	577.61	546.64
5	A1	612.29	612.21	A1	608.10	607.86
6	B2	659.13	659.13	B2	648.83	648.72
ZPE		1198.34	1179.90		1187.27	1168.23
		>> VTZ(-PP):			>> AVTZ(-PP):	
1	B2	148.08	142.09	B2	145.55	140.11
2	A1	195.91	195.69	A1	193.61	193.54
3	B1	212.38	211.88	B1	211.12	210.96
4	A1	614.86	582.37	A1	615.95	584.68
5	A1	623.02	622.56	A1	621.39	619.74
6	B2	665.05	665.00	B2	659.15	659.15
ZPE		1229.65	1209.80		1223.39	1204.09

Quantum-Chemical Results for the Molecule AgF(OF)

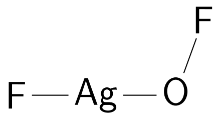

Results for F-Ag-O-F X 2-A"
===========================

Open-shell system, Ag ECP28MDF, 45 electrons
Electron configuration:
- closed-shell part: a’ 1-17, a" 1-5
- open-shell part: (6a")1

Type and number of normal modes: A’ 1-5, A" 1

>> B3LYP [GAUSSIAN]:
Optimized structural parameters, associated total energy, dipole moment:

BASIS	AG-O/ANG	O-F/ANG	AG-F/ANG	AG-O-F/DEG	F-AG-O/DEG	TAU/DEG
AVTZ(-PP)	1.99085	1.38915	1.93056	114.196	176.656	180.000

BASIS	EDFT/AU	MU/D
AVTZ(-PP)	-421.84561590	2.7563

Normal mode analysis (harmonic frequencies, absolute IR intensities in km/mol):

>> AVTZ(-PP):

Vibration Nr		WE*CM [16O/107AG]	[18O/107AG]	[16O/109AG]	[18O/109AG]
1	A"	85.26 (11.43)	83.24 (11.48)	85.06 (11.38)	83.03 (11.43)
2	A’	103.83 (8.37)	103.81 (8.37)	103.64 (8.35)	103.62 (8.35)
3	A’	219.34 (3.43)	216.59 (3.44)	218.96 (3.40)	216.21 (3.40)
4	A’	428.97 (22.83)	409.87 (19.40)	428.89 (22.75)	409.76 (19.30)
5	A’	583.83 (37.86)	580.45 (40.79)	582.62 (37.86)	579.24 (40.80)
6	A’	975.67 (303.47)	946.05 (283.48)	975.67 (303.49)	946.05 (283.50)
ZPE		1198.46	1170.00	1197.42	1168.95

>> RCCSD(T) [CFOUR]:
Optimized structural parameters, associated total energy:

BASIS	AG-O/ANG	O-F/ANG	AG-F/ANG	AG-O-F/DEG	F-AG-O/DEG	TAU/DEG
AVTZ(-PP)	2.05003	1.37191	1.94112	113.432	176.753	180.000

BASIS	ECC/AU
AVTZ(-PP)	-420.91010181

Normal mode analysis (harmonic frequencies, absolute IR intensities in km/mol):

>> AVTZ(-PP):

Vibration Nr		WE*CM [16O/107AG]	[18O/107AG]	[16O/109AG]	[18O/109AG]
1	A"	88.72 (11.99)	87.06 (11.55)	88.51 (11.93)	86.85 (11.49)
2	A’	90.24 (11.05)	90.23 (11.04)	90.07 (11.01)	90.05 (11.00)
3	A’	180.21 (18.25)	177.84 (18.48)	179.88 (18.16)	177.52 (18.38)
4	A’	299.56 (114.23)	285.14 (101.00)	299.46 (114.08)	285.03 (100.84)
5	A’	570.00 (56.48)	568.87 (60.57)	568.95 (56.69)	567.83 (60.80)
6	A’	956.76 (230.23)	928.46 (213.17)	956.76 (230.21)	928.46 (213.16)
ZPE		1092.75	1068.80	1091.82	1067.87

Quantum-Chemical Results for the Molecule Ag(O)F_2

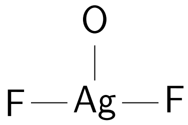


```
Results for O-Ag(-F)2 X 2-B2
============================
Open-shell system, Ag ECP28MDF, 45 electrons
Electron configuration:
- closed-shell part: a1 1-10, a1 1-2, b1 1-4, b2 1-6
-   open-shell part: (7b2)1
Type and number of normal modes: A1 1-3, B1 1, B2 1-2

>> B3LYP [GAUSSIAN]:
Optimized structural parameters, associated total energy, dipole moment:

BASIS        AG-O/ANG  AG-F/ANG  O-AG-F/DEG        EDFT/AU      MU/D
AVTZ(-PP)     1.89779   1.89215      94.352   -421.83623902   1.1391

Normal mode analysis (harmonic frequencies, absolute IR intensities in km/mol):

    Vibration       WE*CM
       Nr           [16O/107AG]        [18O/107AG]        [16O/109AG]        [18O/109AG]

                 >> AVTZ(-PP):
       1      B2    155.32 (  0.02)    149.62 (  0.02)    155.23 (  0.02)    149.53 (  0.02)
       2      A1    183.22 ( 14.29)    182.96 ( 14.24)    182.86 ( 14.23)    182.61 ( 14.19)
       3      B1    194.49 ( 19.59)    194.39 ( 19.48)    193.98 ( 19.47)    192.88 ( 19.36)
       4      A1    516.30 (  0.02)    490.48 (  0.03)    515.72 (  0.02)    489.87 (  0.02)
       5      A1    555.86 (  5.43)    555.80 (  5.43)    555.86 (  5.43)    555.79 (  5.43)
       6      B2    651.53 (133.45)    651.53 (133.45)    649.97 (132.79)    649.97 (132.79)
      ZPE          1128.36            1112.39            1126.81            1110.82


>> RCCSD(T) [CFOUR]:
Optimized structural parameters, associated total energy:

BASIS        AG-O/ANG  AG-F/ANG  O-AG-F/DEG        ECC/AU
AVTZ(-PP)     1.90745   1.87863      92.695   -420.90450190

Normal mode analysis (harmonic frequencies, absolute IR intensities in km/mol):

    Vibration       WE*CM
       Nr           [16O/107AG]        [18O/107AG]        [16O/109AG]        [18O/109AG]

                 >> AVTZ(-PP):
       1      B2    150.39 (  0.53)    144.91 (  0.52)    150.31 (  0.53)    144.83 (  0.52)
       2      A1    189.42 ( 16.72)    189.15 ( 16.70)    189.05 ( 16.56)    188.79 ( 16.63)
       3      B1    196.97 ( 21.61)    196.92 ( 21.56)    196.46 ( 21.49)    196.42 ( 21.43)
       4      A1    497.80 (  0.73)    472.94 (  0.59)    497.24 (  0.75)    472.35 (  0.61)
       5      A1    576.11 (  5.86)    576.00 (  5.76)    576.11 (  5.86)    576.00 (  5.75)
       6      B2    672.09 (152.26)    672.09 (152.26)    670.47 (151.55)    670.47 (151.56)
      ZPE          1141.39            1126.01            1139.82            1124.43
```

Quantum-Chemical Results for the Molecule CuF(OF)

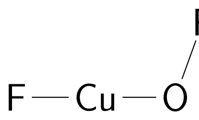


```
Results for F-Cu-O-F X 2-A"
===========================
Open-shell system, Cu ECP10MDF, 45 electrons
Electron configuration:
- closed-shell part: a' 1-17, a" 1-5
-   open-shell part: (6a")1
Type and number of normal modes: A' 1-5, A" 1

>> B3LYP [GAUSSIAN]:
Optimized structural parameters, associated total energy, dipole moment:

BASIS        CU-O/ANG   O-F/ANG  CU-F/ANG  CU-O-F/DEG  F-CU-O/DEG  TAU/DEG
AVTZ(-PP)     1.75198   1.42234   1.71840     109.918     177.021  180.000

BASIS              EDFT/AU      MU/D
AVTZ(-PP)     -472.27302538   1.0328

Normal mode analysis (harmonic frequencies, absolute IR intensities in km/mol):

    Vibration       WE*CM
       Nr           [16O/63CU]         [18O/63CU]         [16O/65CU]         [18O/65CU]

                 >> AVTZ(-PP):
       1      A"     99.89 ( 21.84)     98.01 ( 21.54)     99.32 ( 21.57)     97.44 ( 21.27)
       2      A'    122.67 (  9.98)    122.53 (  9.92)    122.26 (  9.94)    122.13 (  9.89)
       3      A'    268.78 (  7.80)    265.54 (  7.70)    267.54 (  7.62)    264.28 (  7.52)
       4      A'    579.85 (  1.57)    558.65 (  0.50)    579.78 (  1.52)    558.48 (  0.45)
       5      A'    744.15 (130.73)    737.47 (129.75)    740.16 (128.89)    733.50 (127.87)
       6      A'    949.78 ( 80.17)    919.71 ( 76.08)    949.64 ( 81.01)    919.57 ( 76.96)
     ZPE           1382.56            1350.96            1379.35            1347.70


>> RCCSD(T) [CFOUR]:
Optimized structural parameters, associated total energy:

BASIS        CU-O/ANG   O-F/ANG  CU-F/ANG  CU-O-F/DEG  F-CU-O/DEG  TAU/DEG
AVTZ(-PP)     1.73462   1.45001   1.70555     106.081     177.670  180.000

BASIS              ECC/AU
AVTZ(-PP)     -471.20240724

Normal mode analysis (harmonic frequencies, absolute IR intensities in km/mol):

    Vibration       WE*CM
       Nr           [16O/63CU]         [18O/63CU]         [16O/65CU]         [18O/65CU]

                 >> AVTZ(-PP):
       1      A"     97.78 ( 24.31)     96.04 ( 23.46)     97.22 ( 24.04)     95.47 ( 23.18)
       2      A'    121.00 (  9.96)    120.82 (  9.89)    120.64 (  9.93)    120.47 (  9.87)
       3      A'    260.97 ( 12.16)    257.87 ( 11.97)    259.65 ( 11.90)    256.55 ( 11.71)
       4      A'    601.95 (  1.81)    580.87 (  3.80)    601.90 (  1.88)    580.72 (  3.96)
       5      A'    761.69 (197.45)    754.70 (190.49)    758.00 (195.28)    751.09 (188.28)
       6      A'    892.17 (  4.44)    863.12 (  4.47)    891.53 (  4.66)    862.42 (  4.63)
     ZPE           1367.78            1336.71            1364.47            1333.36
```

Quantum-Chemical Results for the Molecule $Cu(O)F_2$

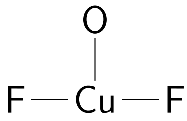

Results for O-Cu(-F)2 X 2-B2
============================
Open-shell system, Cu ECP10MDF, 45 electrons
Electron configuration:
- closed-shell part: a1 1-10, a1 1-2, b1 1-4, b2 1-6
- open-shell part: (7b2)1

Type and number of normal modes: A1 1-3, B1 1, B2 1-2

>> B3LYP [GAUSSIAN]:
Optimized structural parameters, associated total energy, dipole moment:

BASIS	CU-O/ANG	CU-F/ANG	O-CU-F/DEG	EDFT/AU	MU/D
AVTZ(-PP)	1.75860	1.71296	97.008	-472.27105807	0.6057

Normal mode analysis (harmonic frequencies, absolute IR intensities in km/mol):

Vibration Nr		WE*CM [16O/63CU]	[18O/63CU]	[16O/65CU]	[18O/65CU]
	>> AVTZ(-PP):				
1	B2	169.93 (0.01)	164.13 (0.01)	169.72 (0.01)	163.91 (0.01)
2	A1	200.85 (18.61)	200.13 (18.57)	200.04 (18.45)	199.36 (18.41)
3	B1	204.92 (30.97)	204.71 (30.81)	203.58 (30.54)	203.37 (30.38)
4	A1	508.11 (5.89)	485.18 (4.57)	506.73 (5.97)	483.68 (4.66)
5	A1	621.81 (5.48)	621.34 (5.91)	621.55 (5.49)	621.11 (5.90)
6	B2	762.35 (132.12)	762.35 (132.12)	757.98 (130.61)	757.98 (130.61)
ZPE		1233.98	1218.91	1229.80	1214.70

>> RCCSD(T) [CFOUR]:
Optimized structural parameters, associated total energy:

BASIS	CU-O/ANG	CU-F/ANG	O-CU-F/DEG	EDFT/AU
AVTZ(-PP)	1.77133	1.70388	95.751	-471.21194368

Normal mode analysis (harmonic frequencies, absolute IR intensities in km/mol):

Vibration Nr		WE*CM [16O/63CU]	[18O/63CU]	[16O/65CU]	[18O/65CU]
	>> AVTZ(-PP):				
1	B2	160.12 (1.12)	154.62 (1.05)	159.93 (1.11)	154.42 (1.04)
2	A1	203.76 (25.53)	203.08 (25.42)	202.92 (25.30)	202.28 (25.20)
3	B1	216.06 (31.33)	215.90 (31.28)	214.67 (30.93)	214.52 (30.89)
4	A1	510.19 (1.94)	487.03 (1.36)	509.05 (2.03)	485.49 (1.44)
5	A1	641.52 (3.83)	641.43 (3.93)	641.39 (3.81)	641.31 (3.90)
6	B2	788.75 (133.38)	788.75 (133.37)	784.22 (131.98)	784.22 (131.97)
ZPE		1260.35	1245.41	1256.09	1241.12
